# Supplementary material for: The impact of social media interventions on eating behaviours and diet in adolescents and young adults: a mixed methods systematic review protocol
Source: BMJ Open. 2024 Apr 25;14(4):e083465. doi: 10.1136/bmjopen-2023-083465 (PMC11057283; doi:10.1136/bmjopen-2023-083465)
Supplement: Supplementary data [file bmjopen-2023-083465supp001.pdf]

BMJ Publishing Group Limited (BMJ) disclaims all liability and responsibility arising from any reliance placed on this supplemental material which has been supplied by the author(s)

Supplemental Materials

The Impact of Social Media Interventions on Eating Behaviours and Diet in Adolescents and Young Adults: A Mixed Methods Systematic Review Protocol

Table of Contents for Supplemental Materials

Supplemental Table S1	Main Systematic Review Search Term Concepts
Supplemental Table S2	Systematic Review Search Strategy
Supplemental Table S3	Eligibility Criteria using PICO for Quantitative Studies
Supplemental Table S4	Eligibility Criteria using PICO for Qualitative Studies

Tang H, *et al. BMJ Open* 2024; 14:e083465. doi: 10.1136/bmjopen-2023-083465

BMJ Publishing Group Limited (BMJ) disclaims all liability and responsibility arising from any reliance placed on this supplemental material which has been supplied by the author(s)

Supplemental Table S1: Main Systematic Review Search Term concepts

**Social Media (1)**	**Healthy Eating (2)**	**Young People (3)**
“Social Media”	Diet* to capture “Dietary Behavio*r”/ “Dietary Pattern”	“Young Adult*” *to capture adult/adults*
“Social Network*” *to capture Social network/social networks/social networking*	Mesh or equivalent of Nutrition	College to capture college students/female/male/etc.
Facebook	“Weight Management”/ “weight loss	“University
Instagram/ Twitter/ TikTok/YouTube/ Discord snapchat OR pinterest OR tumblr OR	Food	“High School*”; “secondary school*”
"sina weibo" OR Wechat	Eating to capture “Eating Behavio*r”, Eating disorders, disordered eating, Healthy Eating	“Adolescen*/ to capture adolescents 13-18 yo
	Intake/consumption	Youth/young people
		Teen*

Supplemental Table S2. Systematic Review Search Strategy

	**ASSIA** (no keywords, article type: scholarly journal)	**Cochrane Library (**Only include trials**)**	**Medline (OVID) 1946-Jan 23,2023**	**Embase (OVID) 1996-current**	**PsycInfo (EBSCO)**	**Web of Science** (No MeSH term)	**Scopus** (No MeSH term)
1	noft("Social Media" OR "Social Network*" OR Facebook OR Instagram OR Twitter OR YouTube OR TikTok OR Discord OR Snapchat OR pinterest OR tumblr OR "sina weibo" OR WeChat)	MeSH descriptor: [Social Media] this term only OR MeSH descriptor: [Online Social Networking] this term only OR (("Social Media" OR "Social Network*" OR Facebook OR Instagram OR Twitter OR YouTube OR TikTok OR Discord OR Snapchat OR pinterest OR tumblr OR "sina weibo" OR WeChat)):ti,ab,kw	Social media/ OR social networking/ or online social networking/ or ("Social Media" OR "Social Network*" OR Facebook OR Instagram OR Twitter OR YouTube OR TikTok OR Discord OR snapchat OR pinterest OR tumblr OR "sina weibo" OR WeChat).kw,ti,ab.	Social media/ or social networking/ or online social networking/ or ("Social Media" or "Social Network*" or Facebook or Instagram or Twitter or YouTube or TikTok or Discord or snapchat or pinterest or tumblr or "sina weibo" or WeChat).kw,ti,ab.	DE "Social Media" OR DE "Online Social Networks" OR DE "Online Community" OR DE "Social Networks" ORTI ("Social Media" OR "Social Network*" OR Facebook OR Instagram OR Twitter OR YouTube OR TikTok OR Discord OR snapchat OR pinterest OR tumblr OR "sina weibo" OR WeChat) OR AB （"Social Media" OR "Social Network*" OR Facebook OR Instagram OR Twitter OR	((TI=("Social Media" OR "Social Network*" OR Facebook OR Instagram OR Twitter OR YouTube OR TikTok OR Discord OR snapchat OR pinterest OR tumblr OR "sina weibo" OR WeChat)) OR AB=("Social Media" OR "Social Network*" OR Facebook OR Instagram OR Twitter OR YouTube	TITLE-ABS-KEY("Social Media" OR "Social Network*" OR Facebook OR Instagram OR Twitter OR YouTube OR TikTok OR Discord OR snapchat OR pinterest OR tumblr OR "sina weibo" OR WeChat)

					YouTube OR TikTok OR Discord OR snapchat OR pinterest OR tumblr OR "sina weibo" OR WeChat) OR KW （"Social Media" OR "Social Network*" OR Facebook OR Instagram OR Twitter OR YouTube OR TikTok OR Discord OR snapchat OR pinterest OR tumblr OR "sina weibo" OR WeChat)	OR TikTok OR Discord OR snapchat OR pinterest OR tumblr OR "sina weibo" OR WeChat)) OR KP=("Social Media" OR "Social Network*" OR Facebook OR Instagram OR Twitter OR YouTube OR TikTok OR Discord OR snapchat OR pinterest OR tumblr OR "sina weibo" OR WeChat)	
2	MAINSUBJECT.EXACT("Nutrition programmes") OR (MAINSUBJECT.EXACT("Diet") OR MAINSUBJECT.EXACT("Nutrition")) OR MAINSUBJECT.EXACT("Weight loss") OR (MAINSUBJECT.EXACT("Food	MeSH descriptor: [Diet] explode all trees OR MeSH descriptor: [Weight Loss] 1 tree(s) exploded OR MeSH descriptor: [Food] explode all trees OR	diet/ or diet, healthy/ or eating/ OR Nutrition Surveys/ or Nutrition Assessment/ OR Weight Reduction	diet/ or diet, healthy/ or eating/ or Nutrition Surveys/ or Nutrition Assessment/ or Weight Reduction Programs/ or	((((DE "Healthy Eating" OR DE "Diets") OR (DE "Nutrition" OR DE "Avoidant/Restrictive Food Intake Disorder")) OR (DE "Weight Loss")) OR	((TI=(Diet* OR Nutrition OR "Weight Management" OR "weight loss" OR Food OR	TITLE-ABS-KEY (Diet* OR Nutrition OR "Weight Management" OR "weight loss" OR

	consumption") OR MAINSUBJECT.EXACT("Food habits")) OR (MAINSUBJECT.EXACT("Eating") OR MAINSUBJECT.EXACT("Compulsive eating") OR MAINSUBJECT.EXACT("Eating behaviour") OR MAINSUBJECT.EXACT("Eating disorders") OR MAINSUBJECT.EXACT("Binge eating")) OR (MAINSUBJECT.EXACT("Energy intake") OR MAINSUBJECT.EXACT("Caloric intake")) OR MAINSUBJECT.EXACT("Food consumption") or noft(Diet* or Nutrition or "Weight Management" or "weight loss" or Food or Eating or intake or consumption)	MeSH descriptor: [Eating] 1 tree(s) exploded OR ((Diet* or Nutrition or "Weight Management" or "weight loss" or Food or Eating or intake or consumption)):ti,ab,kw	Programs/ or Weight Loss/ OR Avoidant Restrictive Food Intake Disorder/ or Food/ or "Diet, Food, and Nutrition"/ or Food Addiction/ OR Eating/ or Binge-Eating Disorder/ or Night Eating Syndrome/ or "Feeding and Eating Disorders"/ or (Diet* or Nutrition or "Weight Management" or "weight loss" or Food or Eating or intake or consumption).kw,ti,ab.	Weight Loss/ or Avoidant Restrictive Food Intake Disorder/ or Food/ or "Diet, Food, and Nutrition"/ or Food Addiction/ or Eating/ or Binge-Eating Disorder/ or Night Eating Syndrome/ or "Feeding and Eating Disorders"/ or (Diet* or Nutrition or "Weight Management" or "weight loss" or Food or Eating or intake or consumption).kw,ti,ab.	(DE "Food Intake")) OR (DE "Eating Behavior" OR DE "Emotional Eating" OR DE "Rumination (Eating)" OR DE "Binge Eating Disorder" OR DE "Binge Eating" OR DE "Purging (Eating Disorders)" OR DE "Eating Disorders" OR DE "Bulimia") OR TI(Diet* OR Nutrition OR “Weight Management” OR “weight loss” OR Food OR Eating OR intake OR consumption) OR AB (Diet* OR Nutrition OR “Weight Management” OR “weight loss” OR Food OR Eating OR intake OR consumption) OR KW (Diet* OR Nutrition OR	Eating OR intake OR consumption)) OR AB=(Diet* OR Nutrition OR “Weight Management” OR “weight loss” OR Food OR Eating OR intake OR consumption)) OR KP=(Diet* OR Nutrition OR “Weight Management” OR “weight loss” OR Food OR Eating OR intake OR consumption)	Food OR Eating OR intake OR consumption)

					“Weight Management” OR “weight loss” OR Food OR Eating OR intake OR consumption)		
3	(MAINSUBJECT.EXACT("Young adults") OR MAINSUBJECT.EXACT("Young adulthood")) OR MAINSUBJECT.EXACT("Junior high schools") OR (MAINSUBJECT.EXACT("Adolescents") OR MAINSUBJECT.EXACT("Adolescence") OR MAINSUBJECT.EXACT("Adolescent boys") or noft("Young Adult*" or College or University or "High School*" or “middle school*” or “Adolescen*“ or youth or "young people" or "Teen*")	MeSH descriptor: [Young Adult] this term only OR MeSH descriptor: [Adolescent] explode all trees OR (("Young Adult*" or College or University or "High School*" or "middle school*" or "Adolescen*" or youth or "young people" or "Teen*")):ti,ab,kw	Young Adult/ or Adolescent/ or Adult/ or Child/ or ("Young Adult*" or College or University or "High School*" or "middle school*" or "Adolescen*" or youth or "young people" or "Teen*").kw,ti,ab.	Young Adult/ or Adolescent/ or Adult/ or Child/ or ("Young Adult*" or College or University or "High School*" or "middle school*" or "Adolescen*" or youth or "young people" or "Teen*").kw,ti,ab .	DE "College Students" OR DE "High School Students" OR DE "Middle School Students" OR DE "Intermediate School Students" OR DE "Middle School Students" OR DE "Intermediate School Students" OR TI ("Young Adult*" OR College OR University OR "High School*" OR “middle school*” or“Adolescen*” or youth OR “young people” OR "Teen*") OR AB ("Young Adult*" OR College OR University OR "High School*" “middle school*”	((TI=("Young Adult*" OR College OR University OR "High School*" OR "middle school*" OR "Adolescen*" OR youth OR “young people” OR "Teen*")) AND AB=(("Young Adult*" OR College OR University OR "High School*" OR "middle school*" OR "Adolescen*" OR youth OR “young people” OR "Teen*")))	TITLE-ABS-KEY ("Young Adult*" OR College OR University OR "High School*" OR “middle school*” OR “Adolescen*” youth OR “young people” OR "Teen*")

					or“Adolescen*” OR youth OR “young people” OR "Teen*") OR KW ("Young Adult*" OR College OR University OR "High School*"“middle school*” or“Adolescen*” OR youth OR “young people” OR "Teen*")	AND KP=(("Young Adult*" OR College OR University OR "High School*" OR "middle school*" OR "Adolescen*" OR youth OR “young people” OR "Teen*"))	

Supplemental Table S3. Eligibility Criteria using PICO (Quantitative studies)

**PICO**	**Inclusion Criteria**	**Exclusion Criteria**
Population	● 13-35 years old:<br>o Mean OR medium age reported within this range.<br>o This is the age reported in the study.<br>o If only an age range is reported, there must be participants that are 13-35 (inclusive) e.g., if age range is 13-20 = include, if age range is 36-40 = exclude.<br>● Can be included with OR without health conditions.<br>● General population<br>● No limitation for BMI or weight (include obesity for now)	● Mean OR median age reported to be above 35 years OR below 13 years.<br>● Age range does not include 13–35-year-old.<br>● If no mean is mentioned, exclude if >50% of the sample are less than 13 OR over 35 years old.<br>● Institutionalized populations e.g., prisons<br>● Target Participants with pre-existing health conditions (e.g., diabetes, eating disorders, post-surgery, cancer survivors, anaemia, food allergies …) OR those selected solely based on a clinical outcome.<br>● Pregnant and lactating women
**Intervention**	● Researcher-led behavioural interventions<br>● Larger scale behaviour change campaigns that were mostly conducted on commercial social media platforms	● Interventions in clinical settings<br>● Interventions not delivered primarily via social media, i.e., the primary function is not social media (e.g. weight loss apps such as WW that have social media function)<br>● Any other social media that are not classified as profile and user-generated content and are not listed above. FOR example, zoom OR social media platforms created specifically for an intervention.
Comparison	● Not necessary to have a control group for interventions.<br>● If have a control group:<br>o standard care/no intervention<br>o traditional intervention (in clinical settings, in-person intervention, etc.)	
Outcomes	**Primary outcome**<br>Changes in diet/dietary behaviours/diet pattern (pre-post intervention) measured using validated questionnaires to assess.<br>● Food variety, food type, SSB consumption etc.<br>- *food frequency questionnaire*<br>● Diet quality	● If the intervention only reported on outcomes that are not listed as the primary outcome:<br>o Alcohol intake<br>o Physical activity<br>o Smoking<br>o Nutrition supplement use

	- The Diet Quality Questionnaire (DQQ)<br>● Overall food consumption, including calorie's OR macro-nutrients<br>- 24-hour Food recall<br>- food diary<br>● Other eating behaviours: binge eating, restrictive eating, overeating, emotional eating.<br>- Eating Attitudes Test - 26 Item (EAT-26) Eating Disorder Examination Questionnaire (EDE-Q)<br>- Eating Disorder Diagnostic Scale (EDDS)<br>* **Only include the paper when primary outcomes are included.**<br><br>**Secondary outcomes:**<br>Energy balance-related outcomes such as Physical activity, Weight or BMI)	o Changes in knowledge about and attitudes toward cooking<br>o Changes in frequency of home cooking and/or food preparation will be excluded.
Study design	• Quantitative studies: Descriptive, Correlational, Causal-Comparative/Quasi-Experimental, and Experimental Research. examples include RCTs.<br>• Language: all languages<br>• Settings: all countries<br>• Year of publication: 2000-now<br>• Only peer-reviewed publications<br>• original peer-reviewed primary research articles will be included.	• Non-peer-reviewed grey literature and unpublished trials will be excluded.<br>• Literature review (e.g., scoping review, systematic review)

Supplemental Table S4. Eligibility Criteria using PICO (Qualitative studies)

PICO	Inclusion Criteria	Exclusion Criteria
Population	● 13-35 years old:<br>o Mean OR medium age reported within this range.<br>o This is the age reported in the study.<br>o If only an age range is reported, there must be participants that are 13-35 (inclusive) e.g. if age range is 13-20 = include, if age range is 36-40 = exclude.<br>● Can be included with OR without health conditions.<br>● General population<br>● No limitation for BMI or weight	● Mean OR median age reported to be above 35 years OR below 13 years.<br>● Age range does not include 13–35-year-old.<br>● If no mean is mentioned, exclude if >50% of the sample are less than 13 OR over 35 years old.<br>● Institutionalized populations e.g., prisons<br>● Target Participants with pre-existing health conditions (e.g., diabetes, eating disorders, post-surgery, cancer survivors, anaemia…) OR those selected solely based on a clinical outcome.<br>● Pregnant and lactating women
**Intervention**	● Researcher-led behavioural interventions<br>● Larger scale behaviour change campaigns that were mostly conducted on commercial social media platforms	● Interventions in clinical settings<br>● Interventions not delivered primarily via social media, i.e. the primary function is not social media (e.g. weight loss apps such as WW that have social media function)<br>● Any other social media that are not classified as profile and user-generated content and are not listed above. FOR example, zoom OR social media platforms created specifically for an intervention.<br>● Only include Intervention programs (if the study is about social media use only, exclude)
Comparison	● Not necessary to have a control group for interventions<br>● If have a control group:<br>o standard care/no intervention<br>o traditional intervention (in clinical settings, in-person intervention, etc.)	
Outcomes	**Primary outcome**<br>The views and experience in relation to participating in intervention that aims to change diet/dietary behaviours/diet pattern (pre-post intervention)	● attitude for social media<br>● If the intervention only reported on outcomes that are not listed as the primary outcome:<br>o Alcohol intake<br>o Physical activity

	- qualitative feedback on content/features<br>- facilitators and barriers of using the interventions<br>- participants ‘preferences, expectations, feelings about the interventions	o Smoking<br>o Nutrition supplement use
Study design	Qualitative study:<br>Example include studies using focus groups or interviews.<br>• One-on-one interview<br>• Focus Groups<br>• Semi-structured interviews. ...<br>• Ethnographic research:(observe)<br>• Case study research:<br>• Record keeping (makes use of the already existing reliable documents and similar sources of information as the data source.)<br>• Qualitative observation<br>• Observation Notes.<br>• Open-ended survey.<br>• Participant diaries or journals. ...<br><br>• Language: all languages.<br>• Settings: all countries<br>• Year of publication: 2000-now<br>• Only peer-reviewed publications	• Grey literature<br>• Unpublished papers<br>• Literature review (e.g. scoping review, systematic review)
