# Supplementary material for: The impact of social media interventions on eating behaviours and diet in adolescents and young adults: a mixed methods systematic review protocol
Source: BMJ Open. 2024 Apr 25;14(4):e083465. doi: 10.1136/bmjopen-2023-083465 (PMC11057283; doi:10.1136/bmjopen-2023-083465)
Supplement: Author's manuscript [file bmjopen-2023-083465.draft_revisions.pdf]

BMJ Open

BMJ Open is committed to open peer review. As part of this commitment we make the peer review history of every article we publish publicly available.

When an article is published we post the peer reviewers' comments and the authors' responses online. We also post the versions of the paper that were used during peer review. These are the versions that the peer review comments apply to.

The versions of the paper that follow are the versions that were submitted during the peer review process. They are not the versions of record or the final published versions. They should not be cited or distributed as the published version of this manuscript.

BMJ Open is an open access journal and the full, final, typeset and author-corrected version of record of the manuscript is available on our site with no access controls, subscription charges or pay-per-view fees (http://bmjopen.bmj.com).

If you have any questions on BMJ Open's open peer review process please email info.bmjopen@bmj.com

The impact of social media interventions on eating behaviours and diet in adolescents and young adults: a mixed methods systematic review protocol

Journal:	*BMJ Open*
Manuscript ID	bmjopen-2023-083465
Article Type:	Protocol
Date Submitted by the Author:	20-Dec-2023
Complete List of Authors:	Tang, Hao; University of Cambridge School of Clinical Medicine, MRC Epidemiology Unit<br>Spreckley, Marie; University of Cambridge School of Clinical Medicine, MRC Epidemiology Unit<br>van Sluijs, Esther; University of Cambridge School of Clinical Medicine, MRC Epidemiology Unit<br>Ahern, Amy ; University of Cambridge School of Clinical Medicine, MRC Epidemiology Unit<br>Smith, Andrea; University of Cambridge School of Clinical Medicine, MRC Epidemiology Unit
Keywords:	Adolescent, eHealth, Social Media, NUTRITION & DIETETICS, Behavior

SCHOLARONE™
Manuscripts

The impact of social media interventions on eating behaviours and diet in adolescents and young adults: a mixed methods systematic review protocol

Hao Tang[1], Dr Marie Spreckley[1], Dr Esther M F van Sluijs[1], Dr Amy L Ahern[1], Dr Andrea D Smith[1*]*

[1]MRC Epidemiology Unit, University of Cambridge, UK
**denotes shared senior authorship*

Strength and limitations of this study:

- The mixed-methods scope to this systematic review allows for a comprehensive overview of both quantitative and qualitative research to provide a more holistic understanding of the impact, experiences, perspectives, and preferences of young people regarding the impact of social media interventions on their dietary behaviours.
- Examination of behaviour change techniques (BCTs) and their application in the context of interventions will help us to understand the underlying mechanisms of action and guide future behaviour change intervention design.
- This review includes a comprehensive range of eating behaviours outcomes, including disordered eating behaviours in addition to overall food consumption, food variety, diet quality to capture potential unexpected or adverse outcomes on eating behaviours too.
- Inclusion of studies published in all languages can capture diverse populations and cultural differences of the impact of social media interventions on eating behaviours
- Interpretation of a meta-analysis may be limited by the lack of standardization in reporting of eating behaviours in the context of social media-based behaviour change interventions.

Abstract

Introduction Adolescents and young adults are a susceptible population when it comes to healthy eating and dietary behaviours. The increasing use of social media by this age group presents a unique opportunity to promote healthy eating habits. Social media has become a popular platform for promoting health interventions, particularly among young people. However, there is a lack of consensus on the effectiveness of social media interventions in this population. This mixed-methods systematic review aims to synthesize the available evidence on the impact of social media interventions on healthy eating behaviours among young people, their qualitative views and user experiences, and the intervention characteristics, behaviour change theories and techniques used to promote healthy eating.

Methods and Analysis We will conduct a comprehensive search of seven electronic databases, including ASSIA, Cochrane Library, Embase, MEDLINE, PsycINFO, Scopus, and Web of Science. The search strategy will use a combination of Medical Subject Headings (MeSH) terms and keywords covering three domains: social media, eating behaviours, and young people. The search will be limited to peer-reviewed published papers in any language, published from 2000. Three independent reviewers will screen studies based on pre-determined eligibility criteria.

For peer review only

Data will be extracted and analysed using a convergent segregated mixed-methods approach. We will use random-effects meta-analysis or Synthesis Without Meta-analysis (SWiM) for quantitative data, and thematic synthesis for qualitative data. Finally, narrative synthesis using concurrent triangulation will be used to bring together the results of the mixed-method data analysis to provide a comprehensive and integrated understanding of the impact and other features social media interventions. This systematic review will adhere to the Preferred Reporting Items for Systematic Reviews and Meta-Analyses (PRISMA).

Ethics and dissemination Ethical approval is not required since this systematic review will not collect original data. The outcomes of this review will be shared through peer-reviewed publications, conference presentations, and will contribute to the PhD thesis of the primary author.

PROSPERO registration number CRD42023414476

Introduction

Rationale

Developing behaviour change interventions that effectively promote healthy eating habits and positive attitudes to food among adolescents and young adults is a critical public health priority. This age group experiences significant physical, psychological, and social changes, making it an important developmental period to support and establish healthy eating behaviours that have potential to persist into later adulthood [1-3]. However, the majority of adolescents and young adults do not meet the recommended healthy eating guidelines [4, 5] This can have negative consequences for physical and mental health. Suboptimal dietary intake in this age group is linked to elevated risk for the development of chronic diseases such as type 2 diabetes, cardiovascular disease, and certain types of cancer later in life [6-8]. At the same time, the present food environments facilitate access to palatable foods high in saturated fats, salt and free sugar[9]. These often calorically dense foods may be highly processed and widely promoted via sophisticated marketing strategies, often online, targeted at young people [10, 11]. A report by WHO estimated that 340 million adolescents (10-19 years of age) were living with obesity in 2022 [12]. Even in youth, obesity directly increases risk for various Noncommunicable diseases (NCDs), and indirectly contributes to psychological distress [13, 14]. Interventions are warranted that can support healthy eating habits among adolescents and young adults given the far-reaching benefits for both individuals and society.

The strong integration of social media into young people's lifestyles and daily behaviours presents a unique opportunity to transform contemporary approaches to support healthy eating [15-19]. Social media are internet-based applications that allow users to create a unique profile and enable passive and active exchange of text, image or video-based content, although specific features vary by platform [20]. Recent evidence suggested that most young people aged 13-35 years actively use social media. For example, 97% of 16-24-year-olds in the United Kingdom use social media on a weekly basis [21, 22]. Social media platforms are also seen as trusted resources for information, with 52% of young people using social media to seek advice on health issues [23]. Although these platforms provide opportunities for people to engage with each other in

For peer review only

ways that are beneficial, social media can also allow misinformation to proliferate, especially in relation to diet and other wellbeing-related behaviours. Research has shown that social media platforms can pose risks to young people via exposure to inaccurate or inappropriate content [24, 25], increases risk for disordered eating behaviours like binge eating and restrictive eating [26-29] and is associated with health risk behaviours including alcohol, vaping and unhealthy dietary behaviours[30]

It is also important to acknowledge that the global shift towards online platforms during the COVID-19 pandemic has had a significant impact on the way health information is delivered and received [31, 32]. This trend has resulted in an increased acceptance of receiving health advice and interventions through social media platforms [33, 34]. Furthermore, social media platforms have become more sophisticated, allowing for personalized and interactive content that can be tailored to individual users' needs and preferences. This, in turn, has the potential to increase engagement and improve the effectiveness of interventions aimed at promoting healthy eating behaviours. Given these developments, there is a need for updated research to assess the potential of social media interventions as a means of improving dietary behaviours and preventing diet-related diseases in adolescents and young adults.

Social media interventions can be defined as interventions that are conducted mostly through social media platforms (e.g., Facebook, Instagram, YouTube, etc.). Despite the increasing popularity of social media interventions to promote healthy eating habits and diets among adolescents and young adults [35], a comprehensive overview literature is lacking. This will help consolidate the existing evidence on their effectiveness and address several research gaps, especially regarding the exploration of unintended consequences from providing such information online in a digital format. Previous systematic reviews in adjacent topics have mostly focused on the quantitative assessment of the effects of social media interventions on healthy eating behaviours among young people [35, 36]. Important questions remain on the 'how' and on the design of most beneficial delivery approach [17, 37, 38].

There is a limited understanding of the strategies and mechanisms behind how social media influences young people's eating behaviours and diet, and how these processes can be leveraged to promote healthy eating. To our knowledge, the most recent reviews that analysed the behaviour change techniques (BCTs) and content of social media interventions for eating behaviours only included papers from before 2014 only [39]. Systematically reviewing BCTs and explicitly reporting their application in the context of an interventions is essential to understand the mechanisms of effectiveness and guides future intervention design [40].

This mixed-methods systematic review seeks to synthesize existing quantitative and qualitative research to assess the influence of social media interventions on healthy eating among young people, examining their experiences, perspectives, and preferences. The goal is to identify effective intervention components—'active ingredients'—and to understand user satisfaction and long-term efficacy. Additionally, the review will address potential adverse effects, such as weight stigma and disordered eating, with the aim of informing the development of future interventions that balance positive impact with the minimization of negative outcomes.

For peer review only

OBJECTIVES

This mixed-methods systematic review will evaluate existing quantitative and qualitative evidence on social media interventions for healthy eating in young people.

To address this overarching aim, we will:

1) Synthesise quantitative estimates of how social media interventions impact healthy eating behaviours among young people (13-35 years old).

2) Explore qualitative views and user experiences of young people who have participated in social media interventions aimed at promoting healthy eating behaviours.

3) Identify the intervention characteristics, behaviours change theories, and behaviours change techniques employed in social media interventions targeting healthy eating behaviours among young people (13-35 years old).

METHODS AND ANALYSIS

The reporting of this systematic review will adhere to the Preferred Reporting Items for Systematic Reviews and Meta-Analyses (PRISMA) [41]. This protocol has been registered in the International Prospective Register of Systematic Reviews (ID: CRD42023414476).

Literature search strategy

The information sources and search strategy for this mixed-method systematic review will be designed to ensure a comprehensive and systematic search of the available evidence. The following seven databases will be searched:

- ASSIA
- Cochrane Library (CENTRAL)
- Embase
- MEDLINE
- PsycINFO
- Scopus
- Web of Science

The search strategy will be based on a combination of relevant Medical Subject Headings (MeSH) terms and keywords covering three key domains (1) social media AND (2) eating behaviours AND (3) young people. The search terms are listed in Supplemental Table S1. The search strategy will be iteratively tailored to the specific database to ensure that all relevant studies are identified, while avoiding the inclusion of duplicates or irrelevant studies. The search was restricted to papers published after 2000 since most social media platforms used today were developed since then. The search will be conducted without language

For peer review only

restrictions to ensure that all relevant studies are included in the review. Only peer-reviewed published papers will be included. A final search syntax for each electronic database is included in Supplemental Table S2.

In addition to the databases, the reference lists of eligible studies and relevant systematic reviews and meta-analyses will be searched for additional studies that may have been missed in the initial database search. The searches will be re-run prior to the final analysis.

Eligibility criteria

Studies will be selected according to the criteria outlined below (Supplemental Table S3,Table S4).

Table 1. Inclusion and exclusion criteria

Inclusion Criteria	Exclusion Criteria
**Study design**	
• Quantitative, qualitative or mixed-methods studies<br>• No restriction in language of studies or study. Original peer-reviewed primary research articles<br>• Year of publication: 2000 onwards	• Non-peer-reviewed grey literature and unpublished trials<br>• Literature review (e.g., scoping review, systematic review)
**Population**	
• Age: 13-35 years old<br>• Can be included with OR without health conditions as long as the condition doesn't substantially influence diet/eating behaviours	• Institutional populations<br>• Target Participants with pre-existing health conditions<br>• Pregnant or lactating individuals
**Intervention**	
• Researcher-led behavioural interventions or larger scale behaviour change campaigns that were mostly conducted on commercial social media platforms	• Interventions in clinical settings
**Comparison**	
• Not necessary to have a control group for interventions.	
**Outcomes**	
**Only include the paper when primary outcomes are included.**<br>• **Primary outcome**<br>**Quantitative:** Changes in diet/dietary behaviours/diet pattern<br>**Qualitative:** Views and experience in relation to participating in an intervention aimed at changing diet/dietary behaviours/diet pattern	• If the intervention only reported on outcomes that are not listed as the primary outcome, such as Alcohol intake, smoking, nutrition supplement use, changes in knowledge about and attitudes toward eating and so on.

For peer review only

• **Secondary outcomes:** Energy balance-related outcomes/behaviours such as physical activity, body weight or BMI	

Study designs

We will include quantitative, qualitative and mixed-methods studies. Eligible quantitative studies will include experimental and observational studies, such as randomised controlled trials, cluster randomised controlled trials, quasi-randomised trials, before and after (pre-post) studies, prospective and retrospective studies. We will also include qualitative studies that applied focus groups or one-on-one interview approaches. Only original peer-reviewed primary research articles will be included. No restrictions on language will be set and only studies published after 2000 will be included. This is to ensure the systematic review captures the most relevant data within the context of the contemporary social media landscape and its potential to impact on eating behaviours and diet of adolescents and young adults.

Participants

The population of interest is adolescents and young adults between 13-35 years of age. The eligible age range was chosen based on the following: (1) The lower limit of the defined eligible age range is 13 years because this is the minimum age of setting up an account on commercial social media platforms (e.g., YouTube, Instagram, Twitter, TikTok), (2) 13-35 years comprises the highest percentage of active social media users among all age groups in many countries, such as the UK and the US [42-45], and (3) this age range includes important and formative developmental periods when individuals experience significant changes in their health status and social identities. In most countries, turning 18 is also the age they are legally considered an adult which brings along a new sense of autonomy as well as a wide range of social and legal obligations. With regards to physical health and behaviours, many individuals experience weight gain during the period between 18 and 35 years of age [46, 47].

The present systematic review will exclude studies with a mean or median age reported to be below 13 years or above 35 years. Studies that exclusively involve participants with pre-existing health conditions that may influence dietary behaviours, such as type 2 diabetes, eating disorders, post-surgery, cancer, or anaemia, will be excluded to ensure that generalisability of the results is not biased by these conditions. Studies that selected participants based solely on a clinical outcome, such as allergy risk, may not be relevant to the research question and will also be excluded.

Interventions

The scope of this systematic review will include both researcher-led behavioural interventions and larger scale behaviour change campaigns that were mostly conducted on commercial social

For peer review only

media platforms. These platforms include social media sites such as Facebook, Twitter, Instagram, TikTok and others that are profile based and allow for user-generated content sharing [20]. Social media platforms that are not classified as profile-based with user-generated content will be excluded. For example, platforms like Zoom will be excluded as they are primarily designed for video communications. Similarly, social media platforms created specifically for an intervention will also be excluded as they may have unique characteristics that are not representative of commercial social media platforms. Where intervention delivery may rely on multiple components, only those interventions where the main component (or collectively the largest proportion of components) was delivered via social media will be included.

Comparators

For eligible intervention studies, we will include studies with any type of comparator and those with no comparator (pre-post comparisons). For randomised controlled trails (RCTs), comparators can include standard care/no intervention/waiting-list, and attention-matched controls that do not involve social media.

Outcomes

The main outcomes of the quantitative studies will be changes in dietary behaviours and patterns as measured by questionnaires and/or diet diaries. For example, the measures of overall food consumption (including total daily calories and proportion of macronutrients), food variety, type, and (sugar-sweetened) beverage consumption, diet quality indices, and other eating behaviours such as binge eating, restrictive eating, overeating, and emotional eating. Studies must include at least one of these main outcomes to be included in the systematic review. Interventions only reporting on changes in knowledge about and attitudes towards cooking or changes in frequency of home cooking and/or food preparation habits will be excluded.

For qualitative studies, the main reported outcome must include qualitative feedback, experiences, and perspectives in relation to participating in a social media intervention that aims to change diet, dietary behaviours, and/or diet pattern. This includes qualitative feedback on content/features; facilitators and barriers of using the interventions; preferences, expectations, and feelings in relation to the social media intervention (or social media-delivered components).

Additional outcomes, when reported, will include energy balance-related outcomes such as physical activity, body weight, and body mass index (BMI). These outcomes will provide additional information about the potential impact of the social media interventions on the overall health and wellbeing of young people. Studies will be excluded if the intervention only reported on outcomes that are not listed as the main outcomes, such as alcohol intake, smoking, or nutrition supplement use.

Timing

Outcomes reported at all time points will be extracted. The primary analysis will focus on the outcomes that were collected and reported at the pre-defined end of the intervention.

For peer review only

Settings

Only studies involving participants living in community-based settings will be included. We will exclude studies conducted in clinical settings.

Data management and selection process

The data management and selection process for this review will be performed using a standardized and systematic approach. All studies identified through the search process will be imported into the Endnote X7 reference management software to remove duplicates. The remaining articles will be uploaded into Covidence (www.covidence.org) and more duplicates will be removed. Two independent reviewers will screen all study titles and abstracts in duplicate to determine their eligibility. Any discrepancies will be discussed and, when required, resolved through consultation with an independent reviewer. We will retrieve the full text of all articles identified as potentially relevant and again, two reviewers will screen these to determine eligibility, with discussion to resolve discrepancies and consultation with a third reviewer where necessary. Reasons for exclusion of articles at the full-text screening stage will be recorded.

Data items

Relevant data will be extracted from eligible full text articles using a piloted and standardized data extraction form. Qualitative and quantitative data will be extracted and analysed separately. A subset of extracted and coded data (50% of studies) will be cross-checked by a second reviewer, and any discrepancies resolved in discussion with an independent third reviewer. The following items will be extracted from the eligible quantitative studies:

- General information (e.g., study authors, publication year, country, funding source).
- Study details (e.g., study aim, study design, randomisation method, blinding and allocation concealment).
- Participants information (e.g., demographic characteristics, recruitment methods, sample size, health conditions).
- Attrition/adherence (e.g., total number of participants at baseline and follow-up measurements, differential attrition, attendance, study withdrawal, lost to follow-up).
- Intervention information (e.g., setting, content, behaviour change techniques, intervention duration and frequency, profession delivering the intervention, method of delivery, group or individual delivery).
- Comparator information (e.g., setting, content, intervention duration and frequency, profession delivering the intervention, method of delivery, group or individual delivery).
- Outcomes (e.g., dietary outcome(s) studied, how they were measured, duration of follow-up, statistical analysis, intervention effect sizes).
- Behaviour Change techniques (BCTs), as reported, or described.
- Social media-enabled function or features (e.g., gamification of interface, online community, chatbots)

The following items will be extracted from the eligible qualitative studies:

For peer review only

- General information (e.g., study authors, publication year, country, funding source).
- Study details (e.g., study aim, study design, randomisation method, blinding, and allocation concealment).
- Participants information (e.g., demographic characteristics, sample size, health conditions).
- Intervention information (e.g., setting, content, behaviour change techniques, intervention duration and frequency, method of delivery, group or individual delivery).
- Comparator information, if there are (e.g., setting, content, intervention duration and frequency, profession delivering the intervention, method of delivery, group or individual delivery).

Risk of bias in individual studies

The quality assessment will be conducted at both the study and outcome level, using the Mixed Methods Appraisal Tool (MMAT) [48] for its ability to evaluate diverse study designs. This tool incorporates a spectrum of criteria tailored specifically for the analysis of quantitative, qualitative, and mixed-method research articles. To ensure a rigorous and unbiased assessment, two reviewers will independently appraise the articles and discuss any discrepancies to reach a consensus.

The certainty of evidence for each study will also be assessed using the GRADE criteria [49] to identify the strengths and limitations of the evidence, and it informs the conclusions and recommendations that can be made based on the review findings.

Data synthesis

A ‘convergent segregated approach’ to data synthesis will be applied [50]. We will (1) organize and analyse the qualitative and quantitative data separately; (2) integrate the data using a triangulation method; and (3) conduct narrative synthesis based on research questions and objectives to describe the findings from both the quantitative and qualitative data.

For the quantitative studies reporting on changes in diet, eating behaviours and patterns, if there are sufficient estimates (at least three studies) from independent studies, we will conduct a random-effects meta-analysis to pool and synthesize the results [51]. Based on this model, estimates of standardized mean differences for continuous outcomes and risk ratios for binary outcomes, as well as 95% confidence intervals for each outcome will be provided. Where appropriate, publication bias will also be statistically assessed using funnel plots and the Egger’s test.

However, if there are limited data, or when the studies included in the review have significant heterogeneity in terms of design, population, interventions, or outcomes, Synthesis Without Meta-Analysis (SWiM) will be used [52]. SwiM involves synthesizing and summarizing the findings from individual studies in a narrative or descriptive manner, rather than through statistical pooling of data. This will ensure transparency and reproducibility in the synthesis process. Where

For peer review only

appropriate, a Harvest Plot [53] will be developed and used to visually present quantitative findings.

For the qualitative studies, thematic analysis [54] will be conducted to identify common themes and patterns in the qualitative data [54]. This will allow us to identify deeper understanding of the experiences, perspectives and motivations of young people participating in the intervention This will involve reading and coding all text and tables labelled results (or equivalent), identifying themes, and summarizing the findings. We will focus on identifying participants' preferences, expectations and feelings about the intervention and facilitators and barriers to intervention use**.**

Finally, triangulation [55] will be used to bring together the results of the qualitative and quantitative syntheses to provide a comprehensive and integrated understanding of the impact of social media interventions for promoting healthy eating behaviours among young people. We will present the results of each synthesis on one page and analyse the convergence, complementarity, or discrepancy of the findings. Additionally, a joint display[56] will be employed, visually representing the integrated results to offer a more holistic perspective on the outcomes.

Assessment of heterogeneity

Heterogeneity between the studies in effect measures will be assessed using the I^2 statistic. We will consider an I^2 value > 50% as indicative of substantial heterogeneity. If there are sufficient data to warrant it, we will further conduct sensitivity analyses to explore heterogeneity based on study quality and/or study characteristics (see subgroup analyses). Analyses would be undertaken in R.

Sensitivity analysis

Suitable sensitivity analysis will be informed by the decisions made during the review process and based on consultation with the review team. Sensitivity analysis may be conducted to explore how *Risk of Bias (as indexed by the RoB 2.0 tool)*[57] and participant characteristics effect estimates.

Analysis of subgroups

In the presence of sufficient data, subgroup analysis of prespecified groupings will be performed for the following study characteristics:

- Population characteristics (e.g., age, gender)
- Intervention type (e.g., diet only vs exercise only vs diet and exercise combination, including vs excluding psychological support).
- Intervention duration (e.g., 1 day, 4 weeks, 12 weeks.)

For peer review only

- Frequency and duration of follow-up [15]
- Intervention approach (individual, segmentation, parental involvement)[15, 38]
- Intervention content (weight loss, mental health, combined)
- Intervention delivery method (via social media only, social media combined with text messages, social media combined with in-person contact/workshops)
- Reported or coded behaviour change techniques (e.g., education, goal setting, action planning) using the Behaviour Change Techniques (BCT) Taxonomy v1 classification framework [58].
- Reported functions and features of social media intervention (e.g., gamification (rewards), communities, groups)

Patient and public involvement

Patients and the public will not be involved directly in this study.

Ethics and dissemination

The systematic review does not require ethics clearance since published studies with non-identifiable data will be used. The results of the systematic review will be disseminated via publication in a peer-reviewed journal as well as through conference presentations.

Authors' contributions

HT served as the lead author of the study protocol, responsible for conceptualizing and designing the study, and drafting the manuscript. ADS and ALA provided guidance and feedback throughout the entire process, helping to refine the research question, improve the study design and manuscript. EvS provided input into the development of the protocol and contributes to RoB assessment. MS helped screen abstracts and full texts and refine inclusion criteria. All authors have read and approved the final protocol.

Acknowledgement

We would like to extend our gratitude to the librarian Veronica Phillips from the Cambridge University Medical Library for their invaluable support in designing the literature search strategy. We also acknowledge and thank Jessica Kohli for her assistance with the screening process of this review.

Funding statement

This research did not receive specific funding from an agency in the public, commercial or not-for-profit sectors. ALA, ADS and EvS are funded by the Medical Research Council (grants MC_UU_00006/6 and MC_UU_00006/5)

For peer review only

References

1. Fielding-Singh, P., *You're worth what you eat: Adolescent beliefs about healthy eating, morality and socioeconomic status.* Social Science & Medicine, 2019. **220**: p. 41-48.
2. Winpenny, E.M., et al., *Changes in diet through adolescence and early adulthood: longitudinal trajectories and association with key life transitions.* International Journal of Behavioral Nutrition and Physical Activity, 2018. **15**(1): p. 86.
3. Movassagh, E.Z., et al., *Tracking Dietary Patterns over 20 Years from Childhood through Adolescence into Young Adulthood: The Saskatchewan Pediatric Bone Mineral Accrual Study.* Nutrients, 2017. **9**(9).
4. *Healthy eating: applying All Our Health*. 2023; Available from: https://www.gov.uk/government/publications/healthy-eating-applying-all-our-health/healthy-eating-applying-all-our-health.
5. *Only 1 in 10 Adults Get Enough Fruits or Vegetables*. Available from: https://www.cdc.gov/nccdphp/dnpao/division-information/media-tools/adults-fruits-vegetables.html.
6. Jayedi, A., et al., *Healthy and unhealthy dietary patterns and the risk of chronic disease: an umbrella review of meta-analyses of prospective cohort studies.* British Journal of Nutrition, 2020. **124**(11): p. 1133-1144.
7. Popa-Wagner, A., et al., *Dietary habits, lifestyle factors and neurodegenerative diseases.* Neural regeneration research, 2020. **15**(3): p. 394.
8. Collins, S., et al., *Diet and mental health during emerging adulthood: a systematic review.* Emerging Adulthood, 2022. **10**(3): p. 645-659.
9. Jia, P., *Obesogenic environment and childhood obesity.* Obesity Reviews, 2021. **22**: p. e13158.
10. Folkvord, F. and R.C. Hermans, *Food marketing in an obesogenic environment: a narrative overview of the potential of healthy food promotion to children and adults.* Current Addiction Reports, 2020. **7**: p. 431-436.
11. Harris, J.L., S. Yokum, and F. Fleming-Milici, *Hooked on Junk: Emerging Evidence on How Food Marketing Affects Adolescents' Diets and Long-Term Health.* Current Addiction Reports, 2021. **8**(1): p. 19-27.
12. World Health Organization. *World Obesity Day 2022 – Accelerating action to stop obesity.* 2022 [cited 2023 4 April]; Available from: https://www.who.int/news/item/04-03-2022-world-obesity-day-2022-accelerating-action-to-stop-obesity.
13. NHS. *Statistics on Obesity, Physical Activity and Diet, England, 2020*. Statistics on Obesity, Physical Activity and Diet; Available from: https://digital.nhs.uk/data-and-information/publications/statistical/statistics-on-obesity-physical-activity-and-diet/england-2020.
14. WHO. *Obesity and overweight*. [cited 2023 05 July]; Available from: https://www.who.int/news-room/fact-sheets/detail/obesity-and-overweight.
15. Kouvari, M., et al., *Digital Health Interventions for Weight Management in Children and Adolescents: Systematic Review and Meta-analysis.* Journal of Medical Internet Research, 2022.

For peer review only

16. Hsu, M.S.H., A. Rouf, and M. Allman-Farinelli, *Effectiveness and Behavioral Mechanisms of Social Media Interventions for Positive Nutrition Behaviors in Adolescents: A Systematic Review.* J Adolesc Health, 2018. **63**(5): p. 531-545.
17. Lozano-Chacon, B., V. Suarez-Lledo, and J. Alvarez-Galvez, *Use and Effectiveness of Social-Media-Delivered Weight Loss Interventions among Teenagers and Young Adults: A Systematic Review.* International Journal of Environmental Research and Public Health, 2021. **18**(16): p. 8493.
18. Naderpoor, N., et al., *Editorial: Using technology for healthy lifestyle and weight management.* Frontiers in Endocrinology, 2022. **13**.
19. Montgomery, S.C., et al., *Peer social network processes and adolescent health behaviors: A systematic review.* Preventive Medicine, 2020. **130**: p. 105900.
20. Greenhow, C., S.M. Galvin, and K.B. Staudt Willet, *What Should Be the Role of Social Media in Education?* Policy insights from the behavioral and brain sciences, 2019. **6**(2): p. 178-185.
21. Ofcom, *Children and parents: media use and attitudes report 2022*.
22. *Social Media Use in 2021*. BROOKE AUXIER, MONICA ANDERSON. p. 5.
23. Goodyear, V.A., K.M. Armour, and H. Wood, *Young people and their engagement with health-related social media: new perspectives.* Sport Educ Soc, 2019. **24**(7): p. 673-688.
24. Chen, J. and Y. Wang, *Social Media Use for Health Purposes: Systematic Review.* J Med Internet Res, 2021. **23**(5): p. e17917.
25. Wang, Y., et al., *Systematic literature review on the spread of health-related misinformation on social media.* Social science & medicine, 2019. **240**: p. 112552.
26. Zhang, J., et al., *The Relationship Between SNS Usage and Disordered Eating Behaviors: A Meta-Analysis.* Frontiers in Psychology, 2021. **12**.
27. Teo, N.S.Y. and S.L. Collinson, *Instagram and risk of rumination and eating disorders: An Asian perspective.* Psychology of Popular Media Culture, 2019. **8**(4): p. 491-508.
28. Griffiths, S., et al., *The Contribution of Social Media to Body Dissatisfaction, Eating Disorder Symptoms, and Anabolic Steroid Use Among Sexual Minority Men.* Cyberpsychol Behav Soc Netw, 2018. **21**(3): p. 149-156.
29. Sidani, J.E., et al., *The Association between Social Media Use and Eating Concerns among US Young Adults.* Journal of the Academy of Nutrition and Dietetics, 2016. **116**(9): p. 1465-1472.
30. Purba, A.K., et al., *Social media use and health risk behaviours in young people: systematic review and meta-analysis.* BMJ, 2023. **383**: p. e073552.
31. Alomiry, H., *Information Consumption During Covid - 19.* Open Public Health Journal, 2022. **15**(1): p. e187494452208101.
32. Basch, C.H., et al., *Community Mitigation of COVID-19 and Portrayal of Testing on TikTok: Descriptive Study.* JMIR Public Health Surveill, 2021. **7**(6): p. e29528.
33. Stellefson, M., et al., *Evolving Role of Social Media in Health Promotion: Updated Responsibilities for Health Education Specialists.* Int J Environ Res Public Health, 2020. **17**(4).
34. Trevino, K.M., et al., *Rapid deployment of virtual mind-body interventions during the COVID-19 outbreak: feasibility, acceptability, and implications for future care.* Supportive Care in Cancer, 2021. **29**(2): p. 543-546.

For peer review only

35. Prowse, R. and S. Carsley, *Digital Interventions to Promote Healthy Eating in Children: Umbrella Review.* JMIR pediatrics and parenting, 2021. **4**(4): p. e30160.
36. Hutchesson, M.J., et al., *eHealth interventions targeting nutrition, physical activity, sedentary behavior, or obesity in adults: A scoping review of systematic reviews.* Obesity Reviews, 2021. **22**(10): p. e13295.
37. Goodyear, V.A., et al., *The effect of social media interventions on physical activity and dietary behaviours in young people and adults: a systematic review.* International Journal of Behavioral Nutrition and Physical Activity, 2021. **18**(1).
38. Hunter, R.F., et al., *Social network interventions for health behaviours and outcomes: A systematic review and meta-analysis.* PLOS Medicine, 2019. **16**(9): p. e1002890.
39. Shaw, J.M., et al., *Social media used as a health intervention in adolescent health: A systematic review of the literature.* DIGITAL HEALTH, 2015. **1**(0): p. 205520761558839.
40. Chau, M.M., M. Burgermaster, and L. Mamykina, *The use of social media in nutrition interventions for adolescents and young adults-A systematic review.* Int J Med Inform, 2018. **120**: p. 77-91.
41. Page, M.J., et al., *The PRISMA 2020 statement: an updated guideline for reporting systematic reviews.* Systematic Reviews, 2021. **10**(1).
42. *UK Instagram users by age group 2022* 2023 [cited 2023 12 March]; Available from: https://www.statista.com/statistics/1018012/instagram-users-united-kingdom/?locale=en.
43. *U.S. Instagram users by age group 2022* 2023 [cited 2023 12 March]; Available from: https://www.statista.com/statistics/398166/us-instagram-user-age-distribution/.
44. *U.S. Facebook demographics age 2022*. 2023 [cited 2023 14 March]; Available from: https://www.statista.com/statistics/187549/facebook-distribution-of-users-age-group-usa/.
45. *United Kingdom Facebook users by age and gender 2022* 2023 [cited 2023 14 March]; Available from: https://www.statista.com/statistics/1030057/facebook-users-united-kingdom-age-gender/?locale=en.
46. Pegington, M., D.P. French, and M.N. Harvie, *Why young women gain weight: A narrative review of influencing factors and possible solutions.* Obesity Reviews, 2020. **21**(5): p. e13002.
47. Lanoye, A., A.A. Gorin, and J.G. LaRose, *Young Adults' Attitudes and Perceptions of Obesity and Weight Management: Implications for Treatment Development.* Curr Obes Rep, 2016. **5**(1): p. 14-22.
48. Hong, Q.N., A. Gonzalez-Reyes, and P. Pluye, *Improving the usefulness of a tool for appraising the quality of qualitative, quantitative and mixed methods studies, the Mixed Methods Appraisal Tool (MMAT).* J Eval Clin Pract, 2018. **24**(3): p. 459-467.
49. Guyatt, G.H., et al., *GRADE: an emerging consensus on rating quality of evidence and strength of recommendations.* Bmj, 2008. **336**(7650): p. 924-926.
50. Castro, F.G., et al., *A Methodology for Conducting Integrative Mixed Methods Research and Data Analyses.* J Mix Methods Res, 2010. **4**(4): p. 342-360.
51. Moher, D., et al., *Preferred reporting items for systematic review and meta-analysis protocols (PRISMA-P) 2015 statement.* Systematic Reviews, 2015. **4**(1): p. 1.

For peer review only

52. Campbell, M., et al., *Synthesis without meta-analysis (SWiM) in systematic reviews: reporting guideline.* BMJ, 2020: p. l6890.
53. Ogilvie, D., et al., *The harvest plot: A method for synthesising evidence about the differential effects of interventions.* BMC Medical Research Methodology, 2008. **8**(1): p. 8.
54. Thomas, J. and A. Harden, *Methods for the thematic synthesis of qualitative research in systematic reviews.* BMC Medical Research Methodology, 2008. **8**(1): p. 45.
55. O'Cathain, A., E. Murphy, and J. Nicholl, *Three techniques for integrating data in mixed methods studies.* BMJ, 2010. **341**: p. c4587.
56. Guetterman, T.C., M.D. Fetters, and J.W. Creswell, *Integrating Quantitative and Qualitative Results in Health Science Mixed Methods Research Through Joint Displays.* Ann Fam Med, 2015. **13**(6): p. 554-61.
57. Sterne, J.A.C., et al., *RoB 2: a revised tool for assessing risk of bias in randomised trials.* BMJ, 2019. **366**: p. l4898.
58. Michie, S., et al., *The behavior change technique taxonomy (v1) of 93 hierarchically clustered techniques: building an international consensus for the reporting of behavior change interventions.* Ann Behav Med, 2013. **46**(1): p. 81-95.

Supplemental Materials

The Impact of Social Media Interventions on Eating Behaviours and Diet in Adolescents and Young Adults: A Mixed Methods Systematic Review Protocol

**Table of Contents for Supplemental Materials**	
Supplemental Table S1	Main Systematic Review Search Term Concepts
Supplemental Table S2	Systematic Review Search Strategy
Supplemental Table S3	Eligibility Criteria using PICO for Quantitative Studies
Supplemental Table S4	Eligibility Criteria using PICO for Qualitative Studies

For peer review only

Supplemental Table S1: Main Systematic Review Search Term concepts

**Social Media (1)**	**Healthy Eating (2)**	**Young People (3)**
"Social Media"	Diet* to capture "Dietary Behavio*r"/ "Dietary Pattern"	"Young Adult*" *to capture adult/adults*
"Social Network*" *to capture Social network/social networks/social networking*	Mesh or equivalent of Nutrition	College to capture college students/female/male/etc.
Facebook	"Weight Management"/ "weight loss	"University
Instagram/ Twitter/ TikTok/YouTube/ Discord snapchat OR pinterest OR tumblr OR	Food	"High School*"; "secondary school*"
"sina weibo" OR Wechat	Eating to capture "Eating Behavio*r", Eating disorders, disordered eating, Healthy Eating	"Adolescen*/ to capture adolescents 13-18 yo
	Intake/consumption	Youth/young people
		Teen*

For peer review only

Supplemental Table S2. Systematic Review Search Strategy

	**ASSIA** (no keywords, article type: scholarly journal)	**Cochrane Library (Only include trials)**	**Medline (OVID) 1946-Jan 23,2023**	**Embase (OVID) 1996-current**	**PsycInfo (EBSCO)**	**Web of Science** (No MeSH term)	**Scopus** (No MeSH term)
1	noft("Social Media" OR "Social Network*" OR Facebook OR Instagram OR Twitter OR YouTube OR TikTok OR Discord OR Snapchat OR pinterest OR tumblr OR "sina weibo" OR WeChat)	MeSH descriptor: [Social Media] this term only OR MeSH descriptor: [Online Social Networking] this term only OR (("Social Media" OR "Social Network*" OR Facebook OR Instagram OR Twitter OR YouTube OR TikTok OR Discord OR Snapchat OR pinterest OR tumblr OR "sina weibo" OR WeChat)):ti,ab,kw	Social media/ OR social networking/ or online social networking/ or ("Social Media" OR "Social Network*" OR Facebook OR Instagram OR Twitter OR YouTube OR TikTok OR Discord OR snapchat OR pinterest OR tumblr OR "sina weibo" OR WeChat).kw,ti,ab.	Social media/ or social networking/ or online social networking/ or ("Social Media" or "Social Network*" or Facebook or Instagram or Twitter or YouTube or TikTok or Discord or snapchat or pinterest or tumblr or "sina weibo" or WeChat).kw,ti,ab.	DE "Social Media" OR DE "Online Social Networks" OR DE "Online Community" OR DE "Social Networks" ORTI ("Social Media" OR "Social Network*" OR Facebook OR Instagram OR Twitter OR YouTube OR TikTok OR Discord OR snapchat OR pinterest OR tumblr OR "sina weibo" OR WeChat) OR AB ("Social Media" OR "Social Network*" OR Facebook OR Instagram OR Twitter OR	((TI=("Social Media" OR "Social Network*" OR Facebook OR Instagram OR Twitter OR YouTube OR TikTok OR Discord OR snapchat OR pinterest OR tumblr OR "sina weibo" OR WeChat)) OR AB=("Social Media" OR "Social Network*" OR Facebook OR Instagram OR Twitter OR YouTube	TITLE-ABS-KEY("Social Media" OR "Social Network*" OR Facebook OR Instagram OR Twitter OR YouTube OR TikTok OR Discord OR snapchat OR pinterest OR tumblr OR "sina weibo" OR WeChat)

For peer review only

					YouTube OR TikTok OR Discord OR snapchat OR pinterest OR tumblr OR "sina weibo" OR WeChat) OR KW ("Social Media" OR "Social Network*" OR Facebook OR Instagram OR Twitter OR YouTube OR TikTok OR Discord OR snapchat OR pinterest OR tumblr OR "sina weibo" OR WeChat)	OR TikTok OR Discord OR snapchat OR pinterest OR tumblr OR "sina weibo" OR WeChat)) OR KP=("Social Media" OR "Social Network*" OR Facebook OR Instagram OR Twitter OR YouTube OR TikTok OR Discord OR snapchat OR pinterest OR tumblr OR "sina weibo" OR WeChat)	
2	MAINSUBJECT.EXACT("Nutrition programmes") OR (MAINSUBJECT.EXACT("Diet") OR MAINSUBJECT.EXACT("Nutrition")) OR MAINSUBJECT.EXACT("Weight loss") OR (MAINSUBJECT.EXACT("Food	MeSH descriptor: [Diet] explode all trees OR MeSH descriptor: [Weight Loss] 1 tree(s) exploded OR MeSH descriptor: [Food] explode all trees OR	diet/ or diet, healthy/ or eating/ OR Nutrition Surveys/ or Nutrition Assessment/ OR Weight Reduction	diet/ or diet, healthy/ or eating/ or Nutrition Surveys/ or Nutrition Assessment/ or Weight Reduction Programs/ or	((((DE "Healthy Eating" OR DE "Diets") OR (DE "Nutrition" OR DE "Avoidant/Restrictive Food Intake Disorder")) OR (DE "Weight Loss")) OR	((TI=(Diet* OR Nutrition OR "Weight Management" OR "weight loss" OR Food OR	TITLE-ABS-KEY (Diet* OR Nutrition OR "Weight Management" OR "weight loss" OR

For peer review only

	consumption") OR MAINSUBJECT.EXACT("Food habits")) OR (MAINSUBJECT.EXACT("Eating") OR MAINSUBJECT.EXACT("Compulsive eating") OR MAINSUBJECT.EXACT("Eating behaviour") OR MAINSUBJECT.EXACT("Eating disorders") OR MAINSUBJECT.EXACT("Binge eating")) OR (MAINSUBJECT.EXACT("Energy intake") OR MAINSUBJECT.EXACT("Caloric intake")) OR MAINSUBJECT.EXACT("Food consumption") or noft(Diet* or Nutrition or "Weight Management" or "weight loss" or Food or Eating or intake or consumption)	MeSH descriptor: [Eating] 1 tree(s) exploded OR ((Diet* or Nutrition or "Weight Management" or "weight loss" or Food or Eating or intake or consumption)):ti,ab,kw	Programs/ or Weight Loss/ OR Avoidant Restrictive Food Intake Disorder/ or Food/ or "Diet, Food, and Nutrition"/ or Food Addiction/ OR Eating/ or Binge-Eating Disorder/ or Night Eating Syndrome/ or "Feeding and Eating Disorders"/ or (Diet* or Nutrition or "Weight Management" or "weight loss" or Food or Eating or intake or consumption).kw,ti,ab.	Weight Loss/ or Avoidant Restrictive Food Intake Disorder/ or Food/ or "Diet, Food, and Nutrition"/ or Food Addiction/ or Eating/ or Binge-Eating Disorder/ or Night Eating Syndrome/ or "Feeding and Eating Disorders"/ or (Diet* or Nutrition or "Weight Management" or "weight loss" or Food or Eating or intake or consumption).kw,ti,ab.	(DE "Food Intake")) OR (DE "Eating Behavior" OR DE "Emotional Eating" OR DE "Rumination (Eating)" OR DE "Binge Eating Disorder" OR DE "Binge Eating" OR DE "Purging (Eating Disorders)" OR DE "Eating Disorders" OR DE "Bulimia") OR TI(Diet* OR Nutrition OR "Weight Management" OR "weight loss" OR Food OR Eating OR intake OR consumption) OR AB (Diet* OR Nutrition OR "Weight Management" OR "weight loss" OR Food OR Eating OR intake OR consumption) OR KW (Diet* OR Nutrition OR	Eating OR intake OR consumption)) OR AB=(Diet* OR Nutrition OR "Weight Management" OR "weight loss" OR Food OR Eating OR intake OR consumption)) OR KP=(Diet* OR Nutrition OR "Weight Management" OR "weight loss" OR Food OR Eating OR intake OR consumption)	Food OR Eating OR intake OR consumption)

For peer review only

					"Weight Management" OR "weight loss" OR Food OR Eating OR intake OR consumption)		
3	(MAINSUBJECT.EXACT("Young adults") OR MAINSUBJECT.EXACT("Young adulthood")) OR MAINSUBJECT.EXACT("Junior high schools") OR (MAINSUBJECT.EXACT("Adolescents") OR MAINSUBJECT.EXACT("Adolescence") OR MAINSUBJECT.EXACT("Adolescent boys") or noft("Young Adult*" or College or University or "High School*" or "middle school*" or "Adolescen*" or youth or "young people" or "Teen*")	MeSH descriptor: [Young Adult] this term only OR MeSH descriptor: [Adolescent] explode all trees OR (("Young Adult*" or College or University or "High School*" or "middle school*" or "Adolescen*" or youth or "young people" or "Teen*")):ti,ab,kw	Young Adult/ or Adolescent/ or Adult/ or Child/ or ("Young Adult*" or College or University or "High School*" or "middle school*" or "Adolescen*" or youth or "young people" or "Teen*").kw,ti,ab.	Young Adult/ or Adolescent/ or Adult/ or Child/ or ("Young Adult*" or College or University or "High School*" or "middle school*" or "Adolescen*" or youth or "young people" or "Teen*").kw,ti,ab .	DE "College Students" OR DE "High School Students" OR DE "Middle School Students" OR DE "Intermediate School Students" OR DE "Middle School Students" OR DE "Intermediate School Students" OR TI ("Young Adult*" OR College OR University OR "High School*" OR "middle school*" or"Adolescen*" or youth OR "young people" OR "Teen*") OR AB ("Young Adult*" OR College OR University OR "High School*" "middle school*"	((TI=("Young Adult*" OR College OR University OR "High School*" OR "middle school*" OR "Adolescen*" OR youth OR "young people" OR "Teen*")) AND AB=(("Young Adult*" OR College OR University OR "High School*" OR "middle school*" OR "Adolescen*" OR youth OR "young people" OR "Teen*")))	TITLE-ABS-KEY ("Young Adult*" OR College OR University OR "High School*" OR "middle school*" OR "Adolescen*" youth OR "young people" OR "Teen*")

For peer review only

					or“Adolescen*” OR youth OR “young people” OR "Teen*") OR KW ("Young Adult*" OR College OR University OR "High School*"“middle school*” or“Adolescen*” OR youth OR “young people” OR "Teen*")	AND KP=(("Young Adult*" OR College OR University OR "High School*" OR "middle school*" OR "Adolescen*" " OR youth OR “young people” OR "Teen*"))	

For peer review only

Supplemental Table S3. Eligibility Criteria using PICO (Quantitative studies)

**PICO**	**Inclusion Criteria**	**Exclusion Criteria**
Population	● 13-35 years old:<br>o Mean OR medium age reported within this range.<br>o This is the age reported in the study.<br>o If only an age range is reported, there must be participants that are 13-35 (inclusive) e.g., if age range is 13-20 = include, if age range is 36-40 = exclude.<br>● Can be included with OR without health conditions.<br>● General population<br>● No limitation for BMI or weight (include obesity for now)	● Mean OR median age reported to be above 35 years OR below 13 years.<br>● Age range does not include 13–35-year-old.<br>● If no mean is mentioned, exclude if >50% of the sample are less than 13 OR over 35 years old.<br>● Institutionalized populations e.g., prisons<br>● Target Participants with pre-existing health conditions (e.g., diabetes, eating disorders, post-surgery, cancer survivors, anaemia, food allergies …) OR those selected solely based on a clinical outcome.<br>● Pregnant and lactating women
**Intervention**	● Researcher-led behavioural interventions<br>● Larger scale behaviour change campaigns that were mostly conducted on commercial social media platforms	● Interventions in clinical settings<br>● Interventions not delivered primarily via social media, i.e., the primary function is not social media (e.g. weight loss apps such as WW that have social media function)<br>● Any other social media that are not classified as profile and user-generated content and are not listed above. FOR example, zoom OR social media platforms created specifically for an intervention.
Comparison	● Not necessary to have a control group for interventions.<br>● If have a control group:<br>o standard care/no intervention<br>o traditional intervention (in clinical settings, in-person intervention, etc.)	
Outcomes	**Primary outcome**<br>Changes in diet/dietary behaviours/diet pattern (pre-post intervention) measured using validated questionnaires to assess.<br>● Food variety, food type, SSB consumption etc.<br>- *food frequency questionnaire*<br>● Diet quality	● If the intervention only reported on outcomes that are not listed as the primary outcome:<br>o Alcohol intake<br>o Physical activity<br>o Smoking<br>o Nutrition supplement use

For peer review only

	- The Diet Quality Questionnaire (DQQ)<br>● Overall food consumption, including calorie's OR macro-nutrients<br>- 24-hour Food recall<br>- food diary<br>● Other eating behaviours: binge eating, restrictive eating, overeating, emotional eating.<br>- Eating Attitudes Test - 26 Item (EAT-26) Eating Disorder Examination Questionnaire (EDE-Q)<br>- Eating Disorder Diagnostic Scale (EDDS)<br>* **Only include the paper when primary outcomes are included.**<br><br>**Secondary outcomes:**<br>Energy balance-related outcomes such as Physical activity, Weight or BMI)	o Changes in knowledge about and attitudes toward cooking<br>o Changes in frequency of home cooking and/or food preparation will be excluded.
Study design	• Quantitative studies: Descriptive, Correlational, Causal-Comparative/Quasi-Experimental, and Experimental Research. examples include RCTs.<br>• Language: all languages<br>• Settings: all countries<br>• Year of publication: 2000-now<br>• Only peer-reviewed publications<br>• original peer-reviewed primary research articles will be included.	• Non-peer-reviewed grey literature and unpublished trials will be excluded.<br>• Literature review (e.g., scoping review, systematic review)

For peer review only

Supplemental Table S4. Eligibility Criteria using PICO (Qualitative studies)

**PICO**	**Inclusion Criteria**	**Exclusion Criteria**
Population	• 13-35 years old:<br>o Mean OR medium age reported within this range.<br>o This is the age reported in the study.<br>o If only an age range is reported, there must be participants that are 13-35 (inclusive) e.g. if age range is 13-20 = include, if age range is 36-40 = exclude.<br>• Can be included with OR without health conditions.<br>• General population<br>• No limitation for BMI or weight	• Mean OR median age reported to be above 35 years OR below 13 years.<br>• Age range does not include 13–35-year-old.<br>• If no mean is mentioned, exclude if >50% of the sample are less than 13 OR over 35 years old.<br>• Institutionalized populations e.g., prisons<br>• Target Participants with pre-existing health conditions (e.g., diabetes, eating disorders, post-surgery, cancer survivors, anaemia…) OR those selected solely based on a clinical outcome.<br>• Pregnant and lactating women
**Intervention**	• Researcher-led behavioural interventions<br>• Larger scale behaviour change campaigns that were mostly conducted on commercial social media platforms	• Interventions in clinical settings<br>• Interventions not delivered primarily via social media, i.e. the primary function is not social media (e.g. weight loss apps such as WW that have social media function)<br>• Any other social media that are not classified as profile and user-generated content and are not listed above. FOR example, zoom OR social media platforms created specifically for an intervention.<br>• Only include Intervention programs (if the study is about social media use only, exclude)
Comparison	• Not necessary to have a control group for interventions<br>• If have a control group:<br>o standard care/no intervention<br>o traditional intervention (in clinical settings, in-person intervention, etc.)	
Outcomes	**Primary outcome**<br>The views and experience in relation to participating in intervention that aims to change diet/dietary behaviours/diet pattern (pre-post intervention)	• attitude for social media<br>• If the intervention only reported on outcomes that are not listed as the primary outcome:<br>o Alcohol intake<br>o Physical activity

For peer review only

	- qualitative feedback on content/features<br>- facilitators and barriers of using the interventions<br>- participants ‘preferences, expectations, feelings about the interventions	o Smoking<br>o Nutrition supplement use
Study design	Qualitative study:<br>Example include studies using focus groups or interviews.<br>• One-on-one interview<br>• Focus Groups<br>• Semi-structured interviews. ...<br>• Ethnographic research:(observe)<br>• Case study research:<br>• Record keeping (makes use of the already existing reliable documents and similar sources of information as the data source.)<br>• Qualitative observation<br>• Observation Notes.<br>• Open-ended survey.<br>• Participant diaries or journals. ...<br><br>• Language: all languages.<br>• Settings: all countries<br>• Year of publication: 2000-now<br>• Only peer-reviewed publications	• Grey literature<br>• Unpublished papers<br>• Literature review (e.g. scoping review, systematic review)

For peer review only

BMJ Open

The impact of social media interventions on eating behaviours and diet in adolescents and young adults: a mixed methods systematic review protocol

Journal:	*BMJ Open*
Manuscript ID	bmjopen-2023-083465.R1
Article Type:	Protocol
Date Submitted by the Author:	06-Mar-2024
Complete List of Authors:	Tang, Hao; University of Cambridge School of Clinical Medicine, MRC Epidemiology Unit<br>Spreckley, Marie; University of Cambridge School of Clinical Medicine, MRC Epidemiology Unit<br>van Sluijs, Esther; University of Cambridge School of Clinical Medicine, MRC Epidemiology Unit<br>Ahern, Amy ; University of Cambridge School of Clinical Medicine, MRC Epidemiology Unit<br>Smith, Andrea; University of Cambridge School of Clinical Medicine, MRC Epidemiology Unit
**Primary Subject Heading**:	Nutrition and metabolism
Secondary Subject Heading:	Public health
Keywords:	Adolescent, eHealth, Social Media, NUTRITION & DIETETICS, Behavior

SCHOLARONE™
Manuscripts

The impact of social media interventions on eating behaviours and diet in adolescents and young adults: a mixed methods systematic review protocol

Hao Tang[1], Dr Marie Spreckley[1], Dr Esther M F van Sluijs[1], Dr Amy L Ahern[1], Dr Andrea D Smith[1*]*

[1]MRC Epidemiology Unit, University of Cambridge, UK
**denotes shared senior authorship*

Corresponding Author
Name: Hao Tang
Email: Hao.tang@mrc-epid.cam.ac.uk

Strength and limitations of this study:

- Utilization of a mixed-methods approach to incorporate both quantitative and qualitative data for a comprehensive evaluation.
- Examination of behaviour change techniques (BCTs) within interventions offers insights into underlying mechanisms of action.
- Inclusion of a broad range of eating behaviour outcomes, including disordered eating behaviours, enhances the scope of potential findings.
- Incorporation of studies published in multiple languages helps capture diverse populations and cultural perspectives.
  The interpretation of quantitative result may be limited by the lack of standardized reporting on eating behaviours and small final number of eligible studies.

Abstract

Introduction Adolescents and young adults are a susceptible population when it comes to healthy eating and dietary behaviours. The increasing use of social media by this age group presents a unique opportunity to promote healthy eating habits. Social media has become a popular platform for promoting health interventions, particularly among young people. However, there is a lack of consensus on the effectiveness of social media interventions in this population. This mixed-methods systematic review aims to synthesize the available evidence on the impact of social media interventions on healthy eating behaviours among young people, their qualitative views and user experiences, and the intervention characteristics, behaviour change theories and techniques used to promote healthy eating.

Methods and Analysis We will conduct a comprehensive search of seven electronic databases, including ASSIA, Cochrane Library, Embase, MEDLINE, PsycINFO, Scopus, and Web of Science. The search strategy will use a combination of Medical Subject Headings (MeSH) terms and keywords covering three domains: social media, eating behaviours, and young people. The search will be limited to peer-reviewed published papers in any language, published from 2000. Three independent reviewers will screen studies based on pre-determined eligibility criteria. Data will be extracted and analysed using a convergent segregated mixed-methods approach. We will use random-effects meta-analysis or Synthesis Without Meta-analysis (SWiM) for

For peer review only

quantitative data, and thematic synthesis for qualitative data. Finally, narrative synthesis using concurrent triangulation will be used to bring together the results of the mixed-method data analysis to provide a comprehensive and integrated understanding of the impact and other features social media interventions. This systematic review will adhere to the Preferred Reporting Items for Systematic Reviews and Meta-Analyses (PRISMA).

Ethics and dissemination Ethical approval is not required since this systematic review will not collect original data. The outcomes of this review will be shared through peer-reviewed publications, conference presentations, and will contribute to the PhD thesis of the primary author.

PROSPERO registration number CRD42023414476

Introduction

Rationale

Developing behaviour change interventions that effectively promote healthy eating habits and positive attitudes to food among adolescents and young adults is a critical public health priority. This age group experiences significant physical, psychological, and social changes, making it an important developmental period to support and establish healthy eating behaviours that have potential to persist into later adulthood [1-3]. However, the majority of adolescents and young adults do not meet the recommended healthy eating guidelines [4, 5] This can have negative consequences for physical and mental health. Suboptimal dietary intake in this age group is linked to elevated risk for the development of chronic diseases such as type 2 diabetes, cardiovascular disease, and certain types of cancer later in life [6-8]. At the same time, the present food environments facilitate access to palatable foods high in saturated fats, salt and free sugar[9]. These often calorically dense foods may be highly processed and widely promoted via sophisticated marketing strategies, often online, targeted at young people [10, 11]. A report by WHO estimated that 340 million adolescents (10-19 years of age) were living with obesity in 2022 [12]. Even in youth, obesity directly increases risk for various Noncommunicable diseases (NCDs), and indirectly contributes to psychological distress [13, 14]. Interventions are warranted that can support healthy eating habits among adolescents and young adults given the far-reaching benefits for both individuals and society.

The strong integration of social media into young people's lifestyles and daily behaviours presents a unique opportunity to transform contemporary approaches to support healthy eating [15-19]. Social media are internet-based applications that allow users to create a unique profile and enable passive and active exchange of text, image or video-based content, although specific features vary by platform [20]. Recent evidence suggested that most young people aged 13-35 years actively use social media. For example, 97% of 16-24-year-olds in the United Kingdom use social media on a weekly basis [21, 22]. Social media platforms are also seen as trusted resources for information, with 52% of young people using social media to seek advice on health issues [23]. Although these platforms provide opportunities for people to engage with each other in ways that are beneficial, social media can also allow misinformation to proliferate, especially in relation to diet and other wellbeing-related behaviours. Research has shown that social media

For peer review only

platforms can pose risks to young people via exposure to inaccurate or inappropriate content [24, 25], increases risk for disordered eating behaviours like binge eating and restrictive eating [26-29] and is associated with health risk behaviours including alcohol, vaping and unhealthy dietary behaviours[30]

It is also important to acknowledge that the global shift towards online platforms during the COVID-19 pandemic has had a significant impact on the way health information is delivered and received [31, 32]. This trend has resulted in an increased acceptance of receiving health advice and interventions through social media platforms [33, 34]. Furthermore, social media platforms have become more sophisticated, allowing for personalized and interactive content that can be tailored to individual users' needs and preferences. This, in turn, has the potential to increase engagement and improve the effectiveness of interventions aimed at promoting healthy eating behaviours. Given these developments, there is a need for updated research to assess the potential of social media interventions as a means of improving dietary behaviours and preventing diet-related diseases in adolescents and young adults.

Social media interventions can be defined as interventions that are conducted mostly through social media platforms (e.g., Facebook, Instagram, YouTube, etc.). Despite the increasing popularity of social media interventions to promote healthy eating habits and diets among adolescents and young adults [35], a comprehensive overview literature is lacking. This will help consolidate the existing evidence on their effectiveness and address several research gaps, especially regarding the exploration of unintended consequences from providing such information online in a digital format. Previous systematic reviews in adjacent topics have mostly focused on the quantitative assessment of the effects of social media interventions on healthy eating behaviours among young people [35, 36]. Important questions remain on the 'how' and on the design of most beneficial delivery approach [17, 37, 38].

There is a limited understanding of the strategies and mechanisms behind how social media influences young people's eating behaviours and diet, and how these processes can be leveraged to promote healthy eating. To our knowledge, the most recent reviews that analysed the behaviour change techniques (BCTs) and content of social media interventions for eating behaviours only included papers from before 2014 only [39]. Systematically reviewing BCTs and explicitly reporting their application in the context of an interventions is essential to understand the mechanisms of effectiveness and guides future intervention design [40].

This mixed-methods systematic review seeks to synthesize existing quantitative and qualitative research to assess the influence of social media interventions on healthy eating among young people, examining their experiences, perspectives, and preferences. The goal is to identify effective intervention components—'active ingredients'—and to understand user satisfaction and long-term efficacy. Additionally, the review will address potential adverse effects, such as weight stigma and disordered eating, with the aim of informing the development of future interventions that balance positive impact with the minimization of negative outcomes.

For peer review only

OBJECTIVES

This mixed-methods systematic review will evaluate existing quantitative and qualitative evidence on social media interventions for healthy eating in young people.

To address this overarching aim, we will:

1) Synthesise quantitative estimates of how social media interventions impact healthy eating behaviours among young people (13-35 years old).

2) Explore qualitative views and user experiences of young people who have participated in social media interventions aimed at promoting healthy eating behaviours.

3) Identify the intervention characteristics, behaviours change theories, and behaviours change techniques employed in social media interventions targeting healthy eating behaviours among young people (13-35 years old).

METHODS AND ANALYSIS

The reporting of this systematic review will adhere to the Preferred Reporting Items for Systematic Reviews and Meta-Analyses (PRISMA) [41]. This protocol has been registered in the International Prospective Register of Systematic Reviews (ID: CRD42023414476). The study is planned to begin on 1st March 2023 and end on 1st May 2024.

Literature search strategy

The information sources and search strategy for this mixed-method systematic review will be designed to ensure a comprehensive and systematic search of the available evidence. The following seven databases will be searched:

- ASSIA
- Cochrane Library (CENTRAL)
- Embase
- MEDLINE
- PsycINFO
- Scopus
- Web of Science

The search strategy will be based on a combination of relevant Medical Subject Headings (MeSH) terms and keywords covering three key domains (1) social media AND (2) eating behaviours AND (3) young people. The search terms are listed in Supplemental Table S1. The search strategy will be iteratively tailored to the specific database to ensure that all relevant studies are identified, while avoiding the inclusion of duplicates or irrelevant studies. The search was restricted to papers published after 2000 since most social media platforms used today were developed since then. The search will be conducted without language restrictions to ensure that all relevant studies are included in the review. Only peer-

For peer review only

reviewed published papers will be included. A final search syntax for each electronic database is included in Supplemental Table S2.

In addition to the databases, the reference lists of eligible studies and relevant systematic reviews and meta-analyses will be searched for additional studies that may have been missed in the initial database search. The searches will be re-run prior to the final analysis.

Eligibility criteria

Study selection will adhere to the criteria briefly summarized in Table 1 and detailed in the Supplemental Tables S3 and S4.

Table 1. Inclusion and exclusion criteria

Inclusion Criteria	Exclusion Criteria
**Study design**	
• Quantitative, qualitative or mixed-methods studies<br>• No restriction in language of studies or study. Original peer-reviewed primary research articles<br>• Year of publication: 2000 onwards	• Non-peer-reviewed grey literature and unpublished trials<br>• Literature review (e.g., scoping review, systematic review)
**Population**	
• Age: 13-35 years old<br>• Can be included with OR without health conditions as long as the condition doesn't substantially influence diet/eating behaviours	• Institutional populations<br>• Target Participants with pre-existing health conditions<br>• Pregnant or lactating individuals
**Intervention**	
• Researcher-led behavioural interventions or larger scale behaviour change campaigns that were mostly conducted on commercial social media platforms	• Interventions in clinical settings
**Comparison**	
• Not necessary to have a control group for interventions.	
**Outcomes**	
**Only include the paper when primary outcomes are included.**<br>• **Primary outcome**<br>**<u>Quantitative:</u>** Changes in diet/dietary behaviours/diet pattern<br>**<u>Qualitative:</u>** Views and experience in relation to participating in an intervention aimed at changing diet/dietary behaviours/diet pattern<br>• **Secondary outcomes:** Energy balance-related outcomes/behaviours such as physical activity, body weight or BMI	• If the intervention only reported on outcomes that are not listed as the primary outcome, such as Alcohol intake, smoking, nutrition supplement use, changes in knowledge about and attitudes toward eating and so on.

For peer review only

For peer review only - http://bmjopen.bmj.com/site/about/guidelines.xhtml

Study designs

We will include quantitative, qualitative and mixed-methods studies. Eligible quantitative studies will include experimental and observational studies, such as randomised controlled trials, cluster randomised controlled trials, quasi-randomised trials, before and after (pre-post) studies, prospective and retrospective studies. We will also include qualitative studies that applied focus groups or one-on-one interview approaches. Only original peer-reviewed primary research articles will be included. No restrictions on language will be set and only studies published after 2000 will be included. This is to ensure the systematic review captures the most relevant data within the context of the contemporary social media landscape and its potential to impact on eating behaviours and diet of adolescents and young adults.

Participants

The population of interest is adolescents and young adults between 13-35 years of age. The eligible age range was chosen based on the following: (1) The lower limit of the defined eligible age range is 13 years because this is the minimum age of setting up an account on commercial social media platforms (e.g., YouTube, Instagram, Twitter, TikTok), (2) 13-35 years comprises the highest percentage of active social media users among all age groups in many countries, such as the UK and the US [42-45], and (3) this age range includes important and formative developmental periods when individuals experience significant changes in their health status and social identities. In most countries, turning 18 is also the age they are legally considered an adult which brings along a new sense of autonomy as well as a wide range of social and legal obligations. With regards to physical health and behaviours, many individuals experience weight gain during the period between 18 and 35 years of age [46, 47].

The present systematic review will exclude studies with a mean or median age reported to be below 13 years or above 35 years. Studies that exclusively involve participants with pre-existing health conditions that may influence dietary behaviours, such as type 2 diabetes, eating disorders, post-surgery, cancer, or anaemia, will be excluded to ensure that generalisability of the results is not biased by these conditions. Studies that selected participants based solely on a clinical outcome, such as allergy risk, may not be relevant to the research question and will also be excluded.

Interventions

The scope of this systematic review will include both researcher-led behavioural interventions and larger scale behaviour change campaigns that were mostly conducted on commercial social media platforms. These platforms include social media sites such as Facebook, Twitter, Instagram, TikTok and others that are profile based and allow for user-generated content sharing [20]. Social media platforms that are not classified as profile-based with user-generated content will be excluded. For example, platforms like Zoom will be excluded as they are primarily designed for video communications. Similarly, social media platforms created specifically for an intervention will also be excluded as they may have unique characteristics that are not

For peer review only

representative of commercial social media platforms. Where intervention delivery may rely on multiple components, only those interventions where the main component (or collectively the largest proportion of components) was delivered via social media will be included.

Comparators

For eligible intervention studies, we will include studies with any type of comparator and those with no comparator (pre-post comparisons). For randomised controlled trails (RCTs), comparators can include standard care/no intervention/waiting-list, and attention-matched controls that do not involve social media.

Outcomes

The main outcomes of the quantitative studies will be changes in dietary behaviours and patterns as measured by questionnaires and/or diet diaries. For example, the measures of overall food consumption (including total daily calories and proportion of macronutrients), food variety, type, and (sugar-sweetened) beverage consumption, diet quality indices, and other eating behaviours such as binge eating, restrictive eating, overeating, and emotional eating. Studies must include at least one of these main outcomes to be included in the systematic review. Interventions only reporting on changes in knowledge about and attitudes towards cooking or changes in frequency of home cooking and/or food preparation habits will be excluded.

For qualitative studies, the main reported outcome must include qualitative feedback, experiences, and perspectives in relation to participating in a social media intervention that aims to change diet, dietary behaviours, and/or diet pattern. This includes qualitative feedback on content/features; facilitators and barriers of using the interventions; preferences, expectations, and feelings in relation to the social media intervention (or social media-delivered components).

Additional outcomes, when reported, will include energy balance-related outcomes such as physical activity, body weight, and body mass index (BMI). These outcomes will provide additional information about the potential impact of the social media interventions on the overall health and wellbeing of young people. Studies will be excluded if the intervention only reported on outcomes that are not listed as the main outcomes, such as alcohol intake, smoking, or nutrition supplement use.

Timing

Outcomes reported at all time points will be extracted. The primary analysis will focus on the outcomes that were collected and reported at the pre-defined end of the intervention.

Settings

Only studies involving participants living in community-based settings will be included. We will exclude studies conducted in clinical settings.

Data management and selection process

The data management and selection process for this review will be performed using a

For peer review only

standardized and systematic approach. All studies identified through the search process will be imported into the Endnote X7 reference management software to remove duplicates. The remaining articles will be uploaded into Covidence (www.covidence.org) and more duplicates will be removed. Two independent reviewers will screen all study titles and abstracts in duplicate to determine their eligibility. Any discrepancies will be discussed and, when required, resolved through consultation with an independent reviewer. We will retrieve the full text of all articles identified as potentially relevant and again, two reviewers will screen these to determine eligibility, with discussion to resolve discrepancies and consultation with a third reviewer where necessary. Reasons for exclusion of articles at the full-text screening stage will be recorded.

Data items

Relevant data will be extracted from eligible full text articles using a piloted and standardized data extraction form. Qualitative and quantitative data will be extracted and analysed separately. A subset of extracted and coded data (50% of studies) will be cross-checked by a second reviewer, and any discrepancies resolved in discussion with an independent third reviewer. The following items will be extracted from the eligible quantitative studies:

- General information (e.g., study authors, publication year, country, funding source).
- Study details (e.g., study aim, study design, randomisation method, blinding and allocation concealment).
- Participants information (e.g., demographic characteristics, recruitment methods, sample size, health conditions).
- Attrition/adherence (e.g., total number of participants at baseline and follow-up measurements, differential attrition, attendance, study withdrawal, lost to follow-up).
- Intervention information (e.g., setting, content, behaviour change techniques, intervention duration and frequency, profession delivering the intervention, method of delivery, group or individual delivery).
- Comparator information (e.g., setting, content, intervention duration and frequency, profession delivering the intervention, method of delivery, group or individual delivery).
- Outcomes (e.g., dietary outcome(s) studied, how they were measured, duration of follow-up, statistical analysis, intervention effect sizes).
- Behaviour Change techniques (BCTs), as reported, or described.
- Social media-enabled function or features (e.g., gamification of interface, online community, chatbots)

The following items will be extracted from the eligible qualitative studies:

- General information (e.g., study authors, publication year, country, funding source).
- Study details (e.g., study aim, study design, randomisation method, blinding, and allocation concealment).
- Participants information (e.g., demographic characteristics, sample size, health conditions).
- Intervention information (e.g., setting, content, behaviour change techniques, intervention duration and frequency, method of delivery, group or individual delivery).
- Comparator information, if there are (e.g., setting, content, intervention duration and

For peer review only

frequency, profession delivering the intervention, method of delivery, group or individual delivery).

Risk of bias in individual studies

The quality assessment will be conducted at both the study and outcome level, using the Mixed Methods Appraisal Tool (MMAT) [48] for its ability to evaluate diverse study designs. This tool incorporates a spectrum of criteria tailored specifically for the analysis of quantitative, qualitative, and mixed-method research articles. To ensure a rigorous and unbiased assessment, two reviewers will independently appraise the articles and discuss any discrepancies to reach a consensus.

The certainty of evidence for each study will also be assessed using the GRADE criteria [49] to identify the strengths and limitations of the evidence, and it informs the conclusions and recommendations that can be made based on the review findings.

Data synthesis

A 'convergent segregated approach' to data synthesis will be applied [50]. We will (1) organize and analyse the qualitative and quantitative data separately; (2) integrate the data using a triangulation method; and (3) conduct narrative synthesis based on research questions and objectives to describe the findings from both the quantitative and qualitative data.

For the quantitative studies reporting on changes in diet, eating behaviours and patterns, if there are sufficient estimates (at least three studies) from independent studies, we will conduct a random-effects meta-analysis to pool and synthesize the results [51]. Based on this model, estimates of standardized mean differences for continuous outcomes and risk ratios for binary outcomes, as well as 95% confidence intervals for each outcome will be provided. Where appropriate, publication bias will also be statistically assessed using funnel plots and the Egger's test.

However, if there are limited data, or when the studies included in the review have significant heterogeneity in terms of design, population, interventions, or outcomes, Synthesis Without Meta-Analysis (SWiM) will be used [52]. SwiM involves synthesizing and summarizing the findings from individual studies in a narrative or descriptive manner, rather than through statistical pooling of data. This will ensure transparency and reproducibility in the synthesis process. Where appropriate, a Harvest Plot [53] will be developed and used to visually present quantitative findings.

For the qualitative studies, thematic analysis [54] will be conducted to identify common themes and patterns in the qualitative data [54]. This will allow us to identify deeper understanding of the experiences, perspectives and motivations of young people participating in the intervention

For peer review only

This will involve reading and coding all text and tables labelled results (or equivalent), identifying themes, and summarizing the findings. We will focus on identifying participants' preferences, expectations and feelings about the intervention and facilitators and barriers to intervention use.

Finally, triangulation [55] will be used to bring together the results of the qualitative and quantitative syntheses to provide a comprehensive and integrated understanding of the impact of social media interventions for promoting healthy eating behaviours among young people. We will present the results of each synthesis on one page and analyse the convergence, complementarity, or discrepancy of the findings. Additionally, a joint display[56] will be employed, visually representing the integrated results to offer a more holistic perspective on the outcomes.

Assessment of heterogeneity

Heterogeneity between the studies in effect measures will be assessed using the I^2 statistic. We will consider an I^2 value > 50% as indicative of substantial heterogeneity. If there are sufficient data to warrant it, we will further conduct sensitivity analyses to explore heterogeneity based on study quality and/or study characteristics (see subgroup analyses). Analyses would be undertaken in R.

Sensitivity analysis

Suitable sensitivity analysis will be informed by the decisions made during the review process and based on consultation with the review team. Sensitivity analysis may be conducted to explore how *Risk of Bias (as indexed by the RoB 2.0 tool)*[57] and participant characteristics effect estimates.

Analysis of subgroups

In the presence of sufficient data, subgroup analysis of prespecified groupings will be performed for the following study characteristics:

- Population characteristics (e.g., age, gender)
- Intervention type (e.g., diet only vs exercise only vs diet and exercise combination, including vs excluding psychological support).
- Intervention duration (e.g., 1 day, 4 weeks, 12 weeks.)
- Frequency and duration of follow-up [15]
- Intervention approach (individual, segmentation, parental involvement)[15, 38]
- Intervention content (weight loss, mental health, combined)
- Intervention delivery method (via social media only, social media combined with text messages, social media combined with in-person contact/workshops)

For peer review only

- Reported or coded behaviour change techniques (e.g., education, goal setting, action planning) using the Behaviour Change Techniques (BCT) Taxonomy v1 classification framework [58].
- Reported functions and features of social media intervention (e.g., gamification (rewards), communities, groups)

Patient and public involvement

Patients and the public will not be involved directly in this study.

Ethics and dissemination

The systematic review does not require ethics clearance since published studies with non-identifiable data will be used. The results of the systematic review will be disseminated via publication in a peer-reviewed journal as well as through conference presentations.

Authors' contributions

HT served as the lead author of the study protocol, responsible for conceptualizing and designing the study, and drafting the manuscript. ADS and ALA provided guidance and feedback throughout the entire process, helping to refine the research question, improve the study design and manuscript. EvS provided input into the development of the protocol and contributes to RoB assessment. MS helped screen abstracts and full texts and refine inclusion criteria. All authors have read and approved the final protocol.

Conflict of interest

The authors declare that they have no conflict of interest in relation to this study.

Acknowledgement

We would like to extend our gratitude to the librarian Veronica Phillips from the Cambridge University Medical Library for their invaluable support in designing the literature search strategy. We also acknowledge and thank Jessica Kohli for her assistance with the screening process of this review.

Funding statement

This research did not receive specific funding from an agency in the public, commercial or not-for-profit sectors. ALA, ADS and EvS are funded by the Medical Research Council (grants MC_UU_00006/6 and MC_UU_00006/5)

For peer review only

For peer review only

References

1. Fielding-Singh, P., *You're worth what you eat: Adolescent beliefs about healthy eating, morality and socioeconomic status.* Social Science & Medicine, 2019. **220**: p. 41-48.
2. Winpenny, E.M., et al., *Changes in diet through adolescence and early adulthood: longitudinal trajectories and association with key life transitions.* International Journal of Behavioral Nutrition and Physical Activity, 2018. **15**(1): p. 86.
3. Movassagh, E.Z., et al., *Tracking Dietary Patterns over 20 Years from Childhood through Adolescence into Young Adulthood: The Saskatchewan Pediatric Bone Mineral Accrual Study.* Nutrients, 2017. **9**(9).
4. *Healthy eating: applying All Our Health*. 2023; Available from: https://www.gov.uk/government/publications/healthy-eating-applying-all-our-health/healthy-eating-applying-all-our-health.
5. *Only 1 in 10 Adults Get Enough Fruits or Vegetables*. Available from: https://www.cdc.gov/nccdphp/dnpao/division-information/media-tools/adults-fruits-vegetables.html.
6. Jayedi, A., et al., *Healthy and unhealthy dietary patterns and the risk of chronic disease: an umbrella review of meta-analyses of prospective cohort studies.* British Journal of Nutrition, 2020. **124**(11): p. 1133-1144.
7. Popa-Wagner, A., et al., *Dietary habits, lifestyle factors and neurodegenerative diseases.* Neural regeneration research, 2020. **15**(3): p. 394.
8. Collins, S., et al., *Diet and mental health during emerging adulthood: a systematic review.* Emerging Adulthood, 2022. **10**(3): p. 645-659.
9. Jia, P., *Obesogenic environment and childhood obesity.* Obesity Reviews, 2021. **22**: p. e13158.
10. Folkvord, F. and R.C. Hermans, *Food marketing in an obesogenic environment: a narrative overview of the potential of healthy food promotion to children and adults.* Current Addiction Reports, 2020. **7**: p. 431-436.
11. Harris, J.L., S. Yokum, and F. Fleming-Milici, *Hooked on Junk: Emerging Evidence on How Food Marketing Affects Adolescents' Diets and Long-Term Health.* Current Addiction Reports, 2021. **8**(1): p. 19-27.
12. World Health Organization. *World Obesity Day 2022 – Accelerating action to stop obesity.* 2022 [cited 2023 4 April]; Available from: https://www.who.int/news/item/04-03-2022-world-obesity-day-2022-accelerating-action-to-stop-obesity.
13. NHS. *Statistics on Obesity, Physical Activity and Diet, England, 2020*. Statistics on Obesity, Physical Activity and Diet; Available from: https://digital.nhs.uk/data-and-information/publications/statistical/statistics-on-obesity-physical-activity-and-diet/england-2020.
14. WHO. *Obesity and overweight*. [cited 2023 05 July]; Available from: https://www.who.int/news-room/fact-sheets/detail/obesity-and-overweight.
15. Kouvari, M., et al., *Digital Health Interventions for Weight Management in Children and Adolescents: Systematic Review and Meta-analysis.* Journal of Medical Internet Research, 2022.

For peer review only

16. Hsu, M.S.H., A. Rouf, and M. Allman-Farinelli, *Effectiveness and Behavioral Mechanisms of Social Media Interventions for Positive Nutrition Behaviors in Adolescents: A Systematic Review.* J Adolesc Health, 2018. **63**(5): p. 531-545.

17. Lozano-Chacon, B., V. Suarez-Lledo, and J. Alvarez-Galvez, *Use and Effectiveness of Social-Media-Delivered Weight Loss Interventions among Teenagers and Young Adults: A Systematic Review.* International Journal of Environmental Research and Public Health, 2021. **18**(16): p. 8493.

18. Naderpoor, N., et al., *Editorial: Using technology for healthy lifestyle and weight management.* Frontiers in Endocrinology, 2022. **13**.

19. Montgomery, S.C., et al., *Peer social network processes and adolescent health behaviors: A systematic review.* Preventive Medicine, 2020. **130**: p. 105900.

20. Greenhow, C., S.M. Galvin, and K.B. Staudt Willet, *What Should Be the Role of Social Media in Education?* Policy insights from the behavioral and brain sciences, 2019. **6**(2): p. 178-185.

21. Ofcom, *Children and parents: media use and attitudes report 2022*.

22. *Social Media Use in 2021*. BROOKE AUXIER, MONICA ANDERSON. p. 5.

23. Goodyear, V.A., K.M. Armour, and H. Wood, *Young people and their engagement with health-related social media: new perspectives.* Sport Educ Soc, 2019. **24**(7): p. 673-688.

24. Chen, J. and Y. Wang, *Social Media Use for Health Purposes: Systematic Review.* J Med Internet Res, 2021. **23**(5): p. e17917.

25. Wang, Y., et al., *Systematic literature review on the spread of health-related misinformation on social media.* Social science & medicine, 2019. **240**: p. 112552.

26. Zhang, J., et al., *The Relationship Between SNS Usage and Disordered Eating Behaviors: A Meta-Analysis.* Frontiers in Psychology, 2021. **12**.

27. Teo, N.S.Y. and S.L. Collinson, *Instagram and risk of rumination and eating disorders: An Asian perspective.* Psychology of Popular Media Culture, 2019. **8**(4): p. 491-508.

28. Griffiths, S., et al., *The Contribution of Social Media to Body Dissatisfaction, Eating Disorder Symptoms, and Anabolic Steroid Use Among Sexual Minority Men.* Cyberpsychol Behav Soc Netw, 2018. **21**(3): p. 149-156.

29. Sidani, J.E., et al., *The Association between Social Media Use and Eating Concerns among US Young Adults.* Journal of the Academy of Nutrition and Dietetics, 2016. **116**(9): p. 1465-1472.

30. Purba, A.K., et al., *Social media use and health risk behaviours in young people: systematic review and meta-analysis.* BMJ, 2023. **383**: p. e073552.

31. Alomiry, H., *Information Consumption During Covid - 19.* Open Public Health Journal, 2022. **15**(1): p. e187494452208101.

32. Basch, C.H., et al., *Community Mitigation of COVID-19 and Portrayal of Testing on TikTok: Descriptive Study.* JMIR Public Health Surveill, 2021. **7**(6): p. e29528.

33. Stellefson, M., et al., *Evolving Role of Social Media in Health Promotion: Updated Responsibilities for Health Education Specialists.* Int J Environ Res Public Health, 2020. **17**(4).

34. Trevino, K.M., et al., *Rapid deployment of virtual mind-body interventions during the COVID-19 outbreak: feasibility, acceptability, and implications for future care.* Supportive Care in Cancer, 2021. **29**(2): p. 543-546.

For peer review only

35. Prowse, R. and S. Carsley, *Digital Interventions to Promote Healthy Eating in Children: Umbrella Review.* JMIR pediatrics and parenting, 2021. **4**(4): p. e30160.

36. Hutchesson, M.J., et al., *eHealth interventions targeting nutrition, physical activity, sedentary behavior, or obesity in adults: A scoping review of systematic reviews.* Obesity Reviews, 2021. **22**(10): p. e13295.

37. Goodyear, V.A., et al., *The effect of social media interventions on physical activity and dietary behaviours in young people and adults: a systematic review.* International Journal of Behavioral Nutrition and Physical Activity, 2021. **18**(1).

38. Hunter, R.F., et al., *Social network interventions for health behaviours and outcomes: A systematic review and meta-analysis.* PLOS Medicine, 2019. **16**(9): p. e1002890.

39. Shaw, J.M., et al., *Social media used as a health intervention in adolescent health: A systematic review of the literature.* DIGITAL HEALTH, 2015. **1**(0): p. 205520761558839.

40. Chau, M.M., M. Burgermaster, and L. Mamykina, *The use of social media in nutrition interventions for adolescents and young adults-A systematic review.* Int J Med Inform, 2018. **120**: p. 77-91.

41. Page, M.J., et al., *The PRISMA 2020 statement: an updated guideline for reporting systematic reviews.* Systematic Reviews, 2021. **10**(1).

42. *UK Instagram users by age group 2022* 2023 [cited 2023 12 March]; Available from: https://www.statista.com/statistics/1018012/instagram-users-united-kingdom/?locale=en.

43. *U.S. Instagram users by age group 2022* 2023 [cited 2023 12 March]; Available from: https://www.statista.com/statistics/398166/us-instagram-user-age-distribution/.

44. *U.S. Facebook demographics age 2022*. 2023 [cited 2023 14 March]; Available from: https://www.statista.com/statistics/187549/facebook-distribution-of-users-age-group-usa/.

45. *United Kingdom Facebook users by age and gender 2022* 2023 [cited 2023 14 March]; Available from: https://www.statista.com/statistics/1030057/facebook-users-united-kingdom-age-gender/?locale=en.

46. Pegington, M., D.P. French, and M.N. Harvie, *Why young women gain weight: A narrative review of influencing factors and possible solutions.* Obesity Reviews, 2020. **21**(5): p. e13002.

47. Lanoye, A., A.A. Gorin, and J.G. LaRose, *Young Adults' Attitudes and Perceptions of Obesity and Weight Management: Implications for Treatment Development.* Curr Obes Rep, 2016. **5**(1): p. 14-22.

48. Hong, Q.N., A. Gonzalez-Reyes, and P. Pluye, *Improving the usefulness of a tool for appraising the quality of qualitative, quantitative and mixed methods studies, the Mixed Methods Appraisal Tool (MMAT).* J Eval Clin Pract, 2018. **24**(3): p. 459-467.

49. Guyatt, G.H., et al., *GRADE: an emerging consensus on rating quality of evidence and strength of recommendations.* Bmj, 2008. **336**(7650): p. 924-926.

50. Castro, F.G., et al., *A Methodology for Conducting Integrative Mixed Methods Research and Data Analyses.* J Mix Methods Res, 2010. **4**(4): p. 342-360.

51. Moher, D., et al., *Preferred reporting items for systematic review and meta-analysis protocols (PRISMA-P) 2015 statement.* Systematic Reviews, 2015. **4**(1): p. 1.

For peer review only

52. Campbell, M., et al., *Synthesis without meta-analysis (SWiM) in systematic reviews: reporting guideline.* BMJ, 2020: p. l6890.
53. Ogilvie, D., et al., *The harvest plot: A method for synthesising evidence about the differential effects of interventions.* BMC Medical Research Methodology, 2008. **8**(1): p. 8.
54. Thomas, J. and A. Harden, *Methods for the thematic synthesis of qualitative research in systematic reviews.* BMC Medical Research Methodology, 2008. **8**(1): p. 45.
55. O'Cathain, A., E. Murphy, and J. Nicholl, *Three techniques for integrating data in mixed methods studies.* BMJ, 2010. **341**: p. c4587.
56. Guetterman, T.C., M.D. Fetters, and J.W. Creswell, *Integrating Quantitative and Qualitative Results in Health Science Mixed Methods Research Through Joint Displays.* Ann Fam Med, 2015. **13**(6): p. 554-61.
57. Sterne, J.A.C., et al., *RoB 2: a revised tool for assessing risk of bias in randomised trials.* BMJ, 2019. **366**: p. l4898.
58. Michie, S., et al., *The behavior change technique taxonomy (v1) of 93 hierarchically clustered techniques: building an international consensus for the reporting of behavior change interventions.* Ann Behav Med, 2013. **46**(1): p. 81-95.

For peer review only

For peer review only

Supplemental Materials

The Impact of Social Media Interventions on Eating Behaviours and Diet in Adolescents and Young Adults: A Mixed Methods Systematic Review Protocol

Table of Contents for Supplemental Materials	
Supplemental Table S1	Main Systematic Review Search Term Concepts
Supplemental Table S2	Systematic Review Search Strategy
Supplemental Table S3	Eligibility Criteria using PICO for Quantitative Studies
Supplemental Table S4	Eligibility Criteria using PICO for Qualitative Studies

For peer review only

Supplemental Table S1: Main Systematic Review Search Term concepts

**Social Media (1)**	**<u>Healthy Eating (2)</u>**	**Young People (3)**
“Social Media”	Diet* to capture “Dietary Behavio*r”/ “Dietary Pattern”	“Young Adult*” *to capture adult/adults*
“Social Network*” *to capture Social network/social networks/social networking*	Mesh or equivalent of Nutrition	College to capture college students/female/male/etc.
Facebook	“Weight Management”/ “weight loss	“University
Instagram/ Twitter/ TikTok/YouTube/ Discord snapchat OR pinterest OR tumblr OR	Food	“High School*”; “secondary school*”
"sina weibo" OR Wechat	Eating to capture “Eating Behavio*r”, Eating disorders, disordered eating, Healthy Eating	“Adolescen*/ to capture adolescents 13-18 yo
	Intake/consumption	Youth/young people
		Teen*

For peer review only

Supplemental Table S2. Systematic Review Search Strategy

	**ASSIA**<br>(no keywords, article type: scholarly journal)	**Cochrane Library**<br>**(**Only include trials**)**	**Medline (OVID)**<br>**1946-Jan 23,2023**	**Embase (OVID)**<br>**1996-current**	**PsycInfo (EBSCO)**	**Web of Science**<br>(No MeSH term)	**Scopus**<br>(No MeSH term)
1	noft("Social Media" OR "Social Network*" OR Facebook OR Instagram OR Twitter OR YouTube OR TikTok OR Discord OR Snapchat OR pinterest OR tumblr OR "sina weibo" OR WeChat)	MeSH descriptor: [Social Media] this term only OR MeSH descriptor: [Online Social Networking] this term only OR (("Social Media" OR "Social Network*" OR Facebook OR Instagram OR Twitter OR YouTube OR TikTok OR Discord OR Snapchat OR pinterest OR tumblr OR "sina weibo" OR WeChat)):ti,ab,kw	Social media/ OR social networking/ or online social networking/ or ("Social Media" OR "Social Network*" OR Facebook OR Instagram OR Twitter OR YouTube OR TikTok OR Discord OR snapchat OR pinterest OR tumblr OR "sina weibo" OR WeChat).kw,ti,ab.	Social media/ or social networking/ or online social networking/ or ("Social Media" or "Social Network*" or Facebook or Instagram or Twitter or YouTube or TikTok or Discord or snapchat or pinterest or tumblr or "sina weibo" or WeChat).kw,ti,ab.	DE "Social Media" OR DE "Online Social Networks" OR DE "Online Community" OR DE "Social Networks" ORTI ("Social Media" OR "Social Network*" OR Facebook OR Instagram OR Twitter OR YouTube OR TikTok OR Discord OR snapchat OR pinterest OR tumblr OR "sina weibo" OR WeChat) OR AB ("Social Media" OR "Social Network*" OR Facebook OR Instagram OR Twitter OR	((TI=("Social Media" OR "Social Network*" OR Facebook OR Instagram OR Twitter OR YouTube OR TikTok OR Discord OR snapchat OR pinterest OR tumblr OR "sina weibo" OR WeChat)) OR AB=("Social Media" OR "Social Network*" OR Facebook OR Instagram OR Twitter OR YouTube	TITLE-ABS-KEY("Social Media" OR "Social Network*" OR Facebook OR Instagram OR Twitter OR YouTube OR TikTok OR Discord OR snapchat OR pinterest OR tumblr OR "sina weibo" OR WeChat)

For peer review only

					YouTube OR TikTok OR Discord OR snapchat OR pinterest OR tumblr OR "sina weibo" OR WeChat) OR KW （"Social Media" OR "Social Network*" OR Facebook OR Instagram OR Twitter OR YouTube OR TikTok OR Discord OR snapchat OR pinterest OR tumblr OR "sina weibo" OR WeChat)	OR TikTok OR Discord OR snapchat OR pinterest OR tumblr OR "sina weibo" OR WeChat)) OR KP=("Social Media" OR "Social Network*" OR Facebook OR Instagram OR Twitter OR YouTube OR TikTok OR Discord OR snapchat OR pinterest OR tumblr OR "sina weibo" OR WeChat)	
2	MAINSUBJECT.EXACT("Nutrition programmes") OR (MAINSUBJECT.EXACT("Diet") OR MAINSUBJECT.EXACT("Nutrition")) OR MAINSUBJECT.EXACT("Weight loss") OR (MAINSUBJECT.EXACT("Food	MeSH descriptor: [Diet] explode all trees OR MeSH descriptor: [Weight Loss] 1 tree(s) exploded OR MeSH descriptor: [Food] explode all trees OR	diet/ or diet, healthy/ or eating/ OR Nutrition Surveys/ or Nutrition Assessment/ OR Weight Reduction	diet/ or diet, healthy/ or eating/ or Nutrition Surveys/ or Nutrition Assessment/ or Weight Reduction Programs/ or	((((DE "Healthy Eating" OR DE "Diets") OR (DE "Nutrition" OR DE "Avoidant/Restrictive Food Intake Disorder")) OR (DE "Weight Loss")) OR	((TI=(Diet* OR Nutrition OR “Weight Management” OR “weight loss” OR Food OR	TITLE-ABS-KEY (Diet* OR Nutrition OR “Weight Management” OR “weight loss” OR

	consumption") OR MAINSUBJECT.EXACT("Food habits")) OR (MAINSUBJECT.EXACT("Eating") OR MAINSUBJECT.EXACT("Compulsive eating") OR MAINSUBJECT.EXACT("Eating behaviour") OR MAINSUBJECT.EXACT("Eating disorders") OR MAINSUBJECT.EXACT("Binge eating")) OR (MAINSUBJECT.EXACT("Energy intake") OR MAINSUBJECT.EXACT("Caloric intake")) OR MAINSUBJECT.EXACT("Food consumption") or noft(Diet* or Nutrition or "Weight Management" or "weight loss" or Food or Eating or intake or consumption)	MeSH descriptor: [Eating] 1 tree(s) exploded OR ((Diet* or Nutrition or "Weight Management" or "weight loss" or Food or Eating or intake or consumption)):ti,ab,kw	Programs/ or Weight Loss/ OR Avoidant Restrictive Food Intake Disorder/ or Food/ or "Diet, Food, and Nutrition"/ or Food Addiction/ OR Eating/ or Binge-Eating Disorder/ or Night Eating Syndrome/ or "Feeding and Eating Disorders"/ or (Diet* or Nutrition or "Weight Management" or "weight loss" or Food or Eating or intake or consumption).kw,ti,ab.	Weight Loss/ or Avoidant Restrictive Food Intake Disorder/ or Food/ or "Diet, Food, and Nutrition"/ or Food Addiction/ or Eating/ or Binge-Eating Disorder/ or Night Eating Syndrome/ or "Feeding and Eating Disorders"/ or (Diet* or Nutrition or "Weight Management" or "weight loss" or Food or Eating or intake or consumption).kw,ti,ab.	(DE "Food Intake")) OR (DE "Eating Behavior" OR DE "Emotional Eating" OR DE "Rumination (Eating)" OR DE "Binge Eating Disorder" OR DE "Binge Eating" OR DE "Purging (Eating Disorders)" OR DE "Eating Disorders" OR DE "Bulimia") OR TI(Diet* OR Nutrition OR "Weight Management" OR "weight loss" OR Food OR Eating OR intake OR consumption) OR AB (Diet* OR Nutrition OR "Weight Management" OR "weight loss" OR Food OR Eating OR intake OR consumption) OR KW (Diet* OR Nutrition OR	Eating OR intake OR consumption)) OR AB=(Diet* OR Nutrition OR "Weight Management" OR "weight loss" OR Food OR Eating OR intake OR consumption)) OR KP=(Diet* OR Nutrition OR "Weight Management" OR "weight loss" OR Food OR Eating OR intake OR consumption)	Food OR Eating OR intake OR consumption)

					“Weight Management” OR “weight loss” OR Food OR Eating OR intake OR consumption)		
3	(MAINSUBJECT.EXACT("Young adults") OR MAINSUBJECT.EXACT("Young adulthood")) OR MAINSUBJECT.EXACT("Junior high schools") OR (MAINSUBJECT.EXACT("Adolescents") OR MAINSUBJECT.EXACT("Adolescence") OR MAINSUBJECT.EXACT("Adolescent boys") or noft("Young Adult*" or College or University or "High School*" or “middle school*” or “Adolescen*“ or youth or "young people" or "Teen*")	MeSH descriptor: [Young Adult] this term only OR MeSH descriptor: [Adolescent] explode all trees OR (("Young Adult*" or College or University or "High School*" or "middle school*" or "Adolescen*" or youth or "young people" or "Teen*")):ti,ab,kw	Young Adult/ or Adolescent/ or Adult/ or Child/ or ("Young Adult*" or College or University or "High School*" or "middle school*" or "Adolescen*" or youth or "young people" or "Teen*").kw,ti,ab.	Young Adult/ or Adolescent/ or Adult/ or Child/ or ("Young Adult*" or College or University or "High School*" or "middle school*" or "Adolescen*" or youth or "young people" or "Teen*").kw,ti,ab .	DE "College Students" OR DE "High School Students" OR DE "Middle School Students" OR DE "Intermediate School Students" OR DE "Middle School Students" OR DE "Intermediate School Students" OR TI ("Young Adult*" OR College OR University OR "High School*" OR “middle school*” or“Adolescen*” or youth OR “young people” OR "Teen*") OR AB ("Young Adult*" OR College OR University OR "High School*" “middle school*”	((TI=("Young Adult*" OR College OR University OR "High School*" OR "middle school*" OR "Adolescen*" OR youth OR “young people” OR "Teen*")) AND AB=(("Young Adult*" OR College OR University OR "High School*" OR "middle school*" OR "Adolescen*" OR youth OR “young people” OR "Teen*")))	TITLE-ABS-KEY ("Young Adult*" OR College OR University OR "High School*" OR “middle school*” OR “Adolescen*” youth OR “young people” OR "Teen*")

					or“Adolescen*” OR youth OR “young people” OR "Teen*") OR KW ("Young Adult*" OR College OR University OR "High School*"“middle school*” or“Adolescen*” OR youth OR “young people” OR "Teen*")	AND KP=(("Young Adult*" OR College OR University OR "High School*" OR "middle school*" OR "Adolescen*" " OR youth OR “young people” OR "Teen*"))	

For peer review only

Supplemental Table S3. Eligibility Criteria using PICO (Quantitative studies)

<table>
<tr><th>PICO</th><th>Inclusion Criteria</th><th>Exclusion Criteria</th></tr>
<tr><td>Population</td><td>13-35 years old:
Mean OR medium age reported within this range.
This is the age reported in the study.
If only an age range is reported, there must be participants that are 13-35 (inclusive) e.g., if age range is 13-20 = include, if age range is 36-40 = exclude.
Can be included with OR without health conditions.
General population
No limitation for BMI or weight (include obesity for now)</td><td>Mean OR median age reported to be above 35 years OR below 13 years.
Age range does not include 13–35-year-old.
If no mean is mentioned, exclude if >50% of the sample are less than 13 OR over 35 years old.
Institutionalized populations e.g., prisons
Target Participants with pre-existing health conditions (e.g., diabetes, eating disorders, post-surgery, cancer survivors, anaemia, food allergies …) OR those selected solely based on a clinical outcome.
Pregnant and lactating women</td></tr>
<tr><td>Intervention</td><td>Researcher-led behavioural interventions
Larger scale behaviour change campaigns that were mostly conducted on commercial social media platforms</td><td>Interventions in clinical settings
Interventions not delivered primarily via social media, i.e., the primary function is not social media (e.g. weight loss apps such as WW that have social media function)
Any other social media that are not classified as profile and user-generated content and are not listed above. FOR example, zoom OR social media platforms created specifically for an intervention.</td></tr>
<tr><td>Comparison</td><td>Not necessary to have a control group for interventions.
If have a control group:
standard care/no intervention
traditional intervention (in clinical settings, in-person intervention, etc.)</td><td></td></tr>
<tr><td>Outcomes</td><td>Primary outcome
Changes in diet/dietary behaviours/diet pattern (pre-post intervention) measured using validated questionnaires to assess.
Food variety, food type, SSB consumption etc.
food frequency questionnaire
Diet quality</td><td>If the intervention only reported on outcomes that are not listed as the primary outcome:
Alcohol intake
Physical activity
Smoking
Nutrition supplement use</td></tr>
</table>

For peer review only

	- The Diet Quality Questionnaire (DQQ)<br>● Overall food consumption, including calorie's OR macro-nutrients<br>- 24-hour Food recall<br>- food diary<br>● Other eating behaviours: binge eating, restrictive eating, overeating, emotional eating.<br>- Eating Attitudes Test - 26 Item (EAT-26) Eating Disorder Examination Questionnaire (EDE-Q)<br>- Eating Disorder Diagnostic Scale (EDDS)<br>* **Only include the paper when primary outcomes are included.**<br><br>**Secondary outcomes:**<br>Energy balance-related outcomes such as Physical activity, Weight or BMI)	o Changes in knowledge about and attitudes toward cooking<br>o Changes in frequency of home cooking and/or food preparation will be excluded.
Study design	• Quantitative studies: Descriptive, Correlational, Causal-Comparative/Quasi-Experimental, and Experimental Research. examples include RCTs.<br>• Language: all languages<br>• Settings: all countries<br>• Year of publication: 2000-now<br>• Only peer-reviewed publications<br>• original peer-reviewed primary research articles will be included.	• Non-peer-reviewed grey literature and unpublished trials will be excluded.<br>• Literature review (e.g., scoping review, systematic review)

For peer review only

Supplemental Table S4. Eligibility Criteria using PICO (Qualitative studies)

PICO	Inclusion Criteria	Exclusion Criteria
Population	• 13-35 years old:<br>o Mean OR medium age reported within this range.<br>o This is the age reported in the study.<br>o If only an age range is reported, there must be participants that are 13-35 (inclusive) e.g. if age range is 13-20 = include, if age range is 36-40 = exclude.<br>• Can be included with OR without health conditions.<br>• General population<br>• No limitation for BMI or weight	• Mean OR median age reported to be above 35 years OR below 13 years.<br>• Age range does not include 13–35-year-old.<br>• If no mean is mentioned, exclude if >50% of the sample are less than 13 OR over 35 years old.<br>• Institutionalized populations e.g., prisons<br>• Target Participants with pre-existing health conditions (e.g., diabetes, eating disorders, post-surgery, cancer survivors, anaemia…) OR those selected solely based on a clinical outcome.<br>• Pregnant and lactating women
**Intervention**	• Researcher-led behavioural interventions<br>• Larger scale behaviour change campaigns that were mostly conducted on commercial social media platforms	• Interventions in clinical settings<br>• Interventions not delivered primarily via social media, i.e. the primary function is not social media (e.g. weight loss apps such as WW that have social media function)<br>• Any other social media that are not classified as profile and user-generated content and are not listed above. FOR example, zoom OR social media platforms created specifically for an intervention.<br>• Only include Intervention programs (if the study is about social media use only, exclude)
Comparison	• Not necessary to have a control group for interventions<br>• If have a control group:<br>o standard care/no intervention<br>o traditional intervention (in clinical settings, in-person intervention, etc.)	
Outcomes	**Primary outcome**<br>The views and experience in relation to participating in intervention that aims to change diet/dietary behaviours/diet pattern (pre-post intervention)	• attitude for social media<br>• If the intervention only reported on outcomes that are not listed as the primary outcome:<br>o Alcohol intake<br>o Physical activity

For peer review only

	- qualitative feedback on content/features<br>- facilitators and barriers of using the interventions<br>- participants ‘preferences, expectations, feelings about the interventions	o Smoking<br>o Nutrition supplement use
Study design	Qualitative study:<br>Example include studies using focus groups or interviews.<br>• One-on-one interview<br>• Focus Groups<br>• Semi-structured interviews. ...<br>• Ethnographic research:(observe)<br>• Case study research:<br>• Record keeping (makes use of the already existing reliable documents and similar sources of information as the data source.)<br>• Qualitative observation<br>• Observation Notes.<br>• Open-ended survey.<br>• Participant diaries or journals. ...<br><br>• Language: all languages.<br>• Settings: all countries<br>• Year of publication: 2000-now<br>• Only peer-reviewed publications	• Grey literature<br>• Unpublished papers<br>• Literature review (e.g. scoping review, systematic review)

For peer review only
